# Respiratory viruses induce ferroptosis-like features in lung epithelial cells

**DOI:** 10.1128/spectrum.01957-26

**Published:** 2026-08-28

**Authors:** Ana L. Manzano-Covarrubias, Alessandra Tosato, Christina H.T.J. Mol-van der Veen, Philip Ahmadyar, Jai Walia, Manon E. Woest, Jacqueline De Vries-Idema, Anke Huckriede, Amalia M. Dolga, Martina Schmidt, Karim Rafie

**Affiliations:** 1 Department of Molecular Pharmacology, Groningen Research Institute of Pharmacy, Faculty of Science and Engineering, University of Groningen 3647, Groningen, the Netherlands; 2 Groningen Research Institute for Asthma and COPD (GRIAC), University of Groningen, University Medical Center Groningen, Groningen, the Netherlands; 3 Department of Medical Microbiology and Infection Prevention, University of Groningen, University Medical Center Groningen, Groningen, the Netherlands; Regional Centre for Biotechnology, Faridabad, Haryana, India

**Keywords:** influenza A virus, human adenovirus C5, ferroptosis, mitochondria, lipid peroxidation, iron homeostasis

## Abstract

During infection, viruses can modulate various regulated cell death (RCD) mechanisms to evade host immunity and enhance replication. However, less is known about how viruses alter the recently discovered RCD ferroptosis, which is characterized by an iron-dependent accumulation of lipid peroxidation and mitochondrial fragmentation. Influenza A virus (IAV) H1N1 and human adenovirus type C5 (HAdV-C5) are two common causes of respiratory infections of the upper and lower respiratory tracts and can lead to severe illness. While IAV has been shown to induce ferroptosis to support its replication, less is known about whether this is also true for HAdV-C5. Here, we perform a comparative study investigating ferroptosis features during IAV H1N1 and HAdV-C5 infections using alveolar (A549) and bronchial (BEAS-2B) epithelial cells. Our data reveal that HAdV-C5, similar to H1N1, induces lipid peroxidation in a time-dependent manner, which is partially suppressed in the presence of the ferroptosis inhibitor ferrostatin-1. Strikingly, HAdV-C5 infections only lead to changes in ferritin protein levels, which is responsible for iron storage, during late stages of infection. Furthermore, both H1N1 and HAdV-C5 infections trigger a profound mitochondrial network remodeling in A549, BEAS-2B, and primary human epithelial cells, comparable to that induced by the ferroptosis inducer RSL3, with effects varying by cell type and infection stage. These findings suggest that HAdV-C5, just like H1N1, can activate ferroptosis-like processes, highlighting a potential role for lipid peroxidation and mitochondrial alterations in adenoviral infection.

**IMPORTANCE:**

The findings presented here contribute to a deeper understanding of the molecular mechanisms involved in viral infections of the lung epithelium, particularly in the context of virus-induced lung epithelial cell death, a topic of significant relevance to the area of early viral response mechanisms. By elucidating the role of ferroptosis-like hallmarks encompassing lipid peroxidation and mitochondrial dysfunction, our work may inform the development of new therapeutic strategies aimed at enhancing lung epithelial integrity and barrier function, representing the first layer of defense against human pathogens. The topic is of particular importance given the increasing prevalence of antimicrobial resistance. We believe that our research offers significant insights into the field of viral infections and lung disease and would be of great interest to readers in the field of viral biology and respiratory infections.

## INTRODUCTION

Regulated cell death (RCD) is an important host defense mechanism during viral infections, restricting viral spreading and promoting viral clearance. It is also known that viruses can modulate RCD mechanisms to their benefit to promote replication and evade the immune system response (1–3). Different types of RCD, including apoptosis, pyroptosis, and necroptosis, have been extensively studied in the context of viral infection; however, much less is known about ferroptosis, an iron-dependent form of RCD (4–6). Ferroptosis is characterized by several distinct hallmarks. First, the iron-dependent increase in lipid peroxidation results from a dysfunctional System Xc− (Xc−)/Glutathione Peroxidase 4 (GPX4) antioxidant pathway, ultimately reducing the levels of the antioxidant glutathione (GSH) (7). Second, mitochondrial fragmentation, which is characterized by the loss of cristae as well as a smaller and more compact mitochondrial morphology (4, 7, 8). Although the role of mitochondria in ferroptosis is not well defined, a growing body of evidence indicates that they actively participate in ferroptosis by regulating mitochondrial reactive oxygen species (mtROS) production, iron and lipid metabolism, and redox balancing in the cell (9–11). Furthermore, recent studies reported that viral infections can disrupt these mitochondria-linked pathways to modulate ferroptosis (5, 6, 12, 13). However, little information is available regarding whether virus-induced ferroptosis hallmarks are responsible for any changes in mitochondrial morphology.

Influenza A virus (IAV) is an RNA virus with a negative-sense, single-stranded, segmented genome that recognizes and binds to sialic acids widely present on the surface of nasal and bronchial epithelial cells (14). IAV causes annual epidemics of acute respiratory infections and was the cause of four major pandemics (15). Being one of the few viruses in which ferroptosis has been studied, evidence shows that influenza virus modulates the iron metabolism and GPX4 antioxidant axes to induce ferroptosis for its own benefit (16). It was reported that swine influenza virus upregulates transferrin and transferrin receptor expression to promote intracellular iron accumulation and downregulates GPX4 expression, leading to lipid peroxide production and accumulation in alveolar epithelial A549 cells (17). Similarly, H1N1 IAV increased ferrous and total intracellular iron and caused the mitochondria of infected cells to become smaller, with less cristae and compacted mitochondrial membranes in MLE-12 mouse lung epithelial cells (18). Furthermore, H1N1 IAV uses hemagglutinin to form ferritin-nuclear receptor coactivator 4 condensates, promoting ferritinophagy to increase free intracellular iron (19), and uses transferrin receptors to enter the host to enhance its replication (20).

Human adenoviruses (HAdVs) are a large group of non-enveloped, double-stranded DNA viruses, most of which use the coxsackievirus and adenovirus receptor protein present in epithelial tight junctions for viral attachment and entry (21, 22). With over 110 types classified into seven species (A–G), HAdVs are widely spread and are known to cause severe disease in immunocompromised patients (23, 24). The regulation of apoptosis, necrosis, and autophagy by HAdV has been widely studied (25), but whether ferroptosis is a common RCD mechanism across HAdVs remains unclear. HAdV species C (HAdV-C) account for up to 10% of pediatric and up to 7% of adult respiratory infections and have been known to cause epidemics in congregate living spaces (26, 27). HAdV-C5 is among the most common HAdVs associated with human disease, especially in immunocompromised patients (28), but whether or not it also causes ferroptosis-like features has not yet been reported. The adenovirus early region 1A gene has been reported to inhibit the expression of ferritin and sensitize infected cells to oxidative stress (29). Recent research on fowl adenovirus serotype 4, a virus that causes hydropericardium syndrome and liver damage in poultry, reported on the induction of ferroptosis features, including elevated levels of malondialdehyde (MDA), an end product of lipid peroxidation, as well as reduced levels of GSH, reduced GPX4 activity, and a reduced expression of ferritin heavy chain (FTH1) in a chicken hepatocellular carcinoma cell line (LMH) (30). Similar findings have been reported for HAdV D36, which caused an increase in MDA in the liver tissue of embryonated chicken eggs (31) and a decrease in mitochondrial mass in a dose-dependent manner in infected human skeletal muscle cells (32).

In general, as mentioned above, research with a focus on the interaction between ferroptosis and virus infections is limited. Therefore, in this study, we investigated whether HAdV-C5 infection induces ferroptosis-like features in human bronchial (BEAS-2B) and alveolar (A549) lung epithelial cell lines, as well as in primary human airway epithelial cells. As IAVs are among the few viruses in which ferroptosis signaling mechanisms have been studied (6, 16, 17, 19, 20, 33), we used the H1N1 influenza virus as the model virus to determine whether HAdV-C5 is capable of activating this form of RCD. Our findings indicate that HAdV-C5 causes an increase in lipid peroxidation, seemingly leaving the expression of the iron storage protein ferritin unaffected. Importantly, we demonstrate changes in the mitochondrial network in lung epithelial cells caused by both H1N1 and HAdV-C5 infections.

## MATERIALS AND METHODS

### Cell culture and treatment

Adenocarcinomic human alveolar epithelial (A549) cells (ATCC CCL-185) and human bronchial epithelial (BEAS-2B) cells (ATCC CRL-3588) were cultured in Roswell Park Memorial Institute (RPMI)-1640 medium with GlutaMAX Supplement (RPMI 1640 Medium, GlutaMAX Supplement, Cat. #61870044; Gibco, Thermo Fisher Scientific, the Netherlands) supplemented with 10% (vol/vol) heat-inactivated fetal bovine serum (HI-FBS, Hyclone, USA) and 2% (vol/vol) of antibiotics (penicillin-streptomycin 5,000 U/mL, Cat. #11528876, Gibco, Thermo Fisher Scientific, the Netherlands) and kept in a humidified incubator at 37°C and 5% (vol/vol) CO_2_. Cells were detached with trypsin-EDTA (Cat#L0930, Biowest) and seeded in appropriate cell culture plates. During treatment, cells were maintained in starvation media (RPMI-1640 medium with GlutaMAX Supplement with 1% [vol/vol] HI-FBS and 2% [vol/vol] antibiotics) (34, 35).

RAS-selective lethal small molecule 3 (RSL3, #S8155, Selleckchem, USA) was used at a concentration of 10 μM for A549 and 250 nM for BEAS-2B to trigger ferroptosis, unless otherwise indicated. Ferrostatin-1 (Fer-1; #SML0583, Sigma-Aldrich) was used at 5 μM as a ferroptosis inhibitor (19, 36).

Staurosporine (Cat. #15495139, Thermo Scientific Alfa Aesar, the Netherlands) was used at a concentration of 50 nM for A549 cells and at 5 nM for BEAS-2B cells as an apoptosis inducer. Pan-caspase inhibitors Q-VD-Oph (#1135695-98-5, MP Biomedicals, USA) and Z-VAD-FMK (Cat. #S7023, Selleckchem, the Netherlands) were used at a concentration of 10 μM, and caspase-3 inhibitor Z-DEVD-FMK (Cat. #FMK004, R&D Systems, the Netherlands) was used at concentrations of 10, 50, and 100 μM.

### Human airway epithelial cells

Primary human airway epithelial cells (hAECs) were obtained from healthy lung transplant donors post-mortem within 1–8 h after lung donation. Donor selection followed the Eurotransplant guidelines, requiring the absence of primary lung diseases such as asthma or COPD and limiting smoking history to no more than 20 pack-years. Tissue was collected in Krebs–Henseleit buffer (composition in mM: 117.5 NaCl, 5.6 KCl, 1.18 MgSO_4_, 2.5 CaCl_2_, 1.28 NaH_2_PO_4_, 25 NaHCO_3_, and 5.5 glucose) (37). Airway epithelial cells were isolated by enzymatic digestion, then scraped from residual tracheal and main stem bronchial tissue, and cultured as previously described (38, 39). Briefly, cells were grown in pre-coated flasks (fibronectin [10 μg/mL], bovine type I collagen [30 μg/mL], and bovine serum albumin [10 μg/mL] in phosphate-buffered saline [PBS]) (40), cultured in Keratinocyte Serum-Free Medium with Bovine Pituitary Extract and Epidermal Growth Factor (Cat. #17005042, Invitrogen) supplemented with 1 µM isoproterenol (#I6504, Sigma-Aldrich), and maintained at 37°C and 5% (vol/vol) CO_2_ until confluency. During experiments, submerged cells were cultured in bronchial epithelial cell growth medium (BEGM, Cat. #CC-3170, Lonza) and treated with 1 μM of RSL3 or infected with viruses as described below.

Human lung tissue was obtained from lung transplant donors in strict adherence to the Research Code of the UMCG, as stated on https://umcgresearch.org/w/research-code-umcg, as well as national ethical and professional guidelines, including the Code of Conduct for Health Research and the Declaration of Helsinki. Human lung tissue was acquired from extra tissue left over after lung surgeries, which exceeded the amount needed for clinical care purposes. All samples and clinical information were blinded before experiments.

### Viruses and infection

Influenza virus strain A/Puerto Rico/8/34 (H1N1/PR8) was kindly provided by Prof. Dr. Anke Huckriede (Department of Medical Microbiology, University of Groningen, the Netherlands). The virus was propagated in embryonated chicken eggs, purified by sucrose density gradient centrifugation, and titrated on MDCK cells as described (41). Human adenovirus type 5 was purchased from The Gene Vector Core at the Baylor College of Medicine. Virus stocks were prepared by amplification in A549 cells followed by titer determination by plaque assay in A549 cells (42).

At 80% confluence, cells were infected with H1N1 or HAdV-C5 at a multiplicity of infection of 1.0 (MOI 1) in serum-free medium (RPMI-1640 medium with GlutaMAX Supplement, with 2% [vol/vol] antibiotics for A549 and BEAS-2B, and BEGM medium for hAECs) and incubated for 1 h with H1N1 and 2 h with HAdV-C5 to allow the adsorption of the virus; then, cells were washed three times with PBS, and starvation medium was added. Infected cells were incubated for the desired time in a humidified incubator at 37°C and 5% (vol/vol) CO_2_.

### Immunofluorescence staining of viral proteins

A549 and BEAS-2B cells were seeded at 20,000 cells/well onto 48-well plates and infected with influenza virus (H1N1/PR8) as described above. After 0, 4, 6, 8, 18, and 24 h post-infection (hpi), cells were washed with PBS and fixed with 4% paraformaldehyde (PFA) solution at room temperature (RT) for 15 min. After washing three more times with PBS, cells were incubated overnight at 4°C in blocking buffer (0.1% [wt/vol] saponin and 1% [wt/vol] bovine serum albumin in PBS). On the following day, cells were stained with primary antibody against influenza A nucleoprotein (NP) (Cat. #MAB8257, Millipore, 1:750 in blocking buffer) for 1 h at RT. Following incubation, cells were washed three times with blocking buffer and then incubated with Alexa Fluor 568-conjugated donkey anti-mouse IgG (H + L) secondary antibody (Cat. #A10037, Invitrogen) for 45 min at RT (43).

A549 and BEAS-2B cells were seeded at 20,000 cells/well onto 48-well plates and infected with HAdV-C5 virus as described above. After 0, 24, 48, 72, and 96 hpi, cells were washed with PBS, fixed with ice-cold methanol, and incubated at −20°C for 10 min. Methanol was removed, and primary antibody against adenovirus hexon protein (Cat. #MAB8052, Millipore, 1:500 in PBS) was added and incubated for 45 min at RT. After incubation, cells were washed three times with PBS and incubated with Alexa Fluor 568-conjugated donkey anti-mouse IgG (H + L) secondary antibody (Cat. #A10037, Invitrogen) for 30 min at RT (44).

After incubation with secondary antibody, cells were washed three times with PBS, and nuclei were stained with DAPI (Cat. #ab104139, Abcam). Fluorescence was detected on a Nikon Inverted Research Fluorescence Microscope ECLIPSE Ti2-E at a constant exposure of 100 ms. At least five images of each well were captured at a nominal magnification of 20×. H1N1- and HAdV-C5-infected cells were quantified by counting nucleoprotein-positive (NP^+^) cells and hexon protein-positive (Hexon^+^) cells, respectively, and DAPI-positive (DAPI^+^) cells using ImageJ (45). Results are shown as a percentage of NP^+^/DAPI^+^ or Hexon^+^/DAPI^+^ cells.

### Cell morphology

After the desired time post-infection or 24-h treatment with RSL3, cells were washed with PBS and fixed with 4% PFA solution at RT for 15 min. Cells were washed three more times with PBS and imaged using a Nikon Eclipse Ti-S microscope at a nominal magnification of 10×.

### Cell metabolic activity assay

Cell metabolic activity was measured using the 3-(4,5-Dimethyl-2-thiazolyl)-2,5-diphenyl-2H-tetrazolium bromide (MTT) assay (#M5655, Sigma-Aldrich, the Netherlands). A549 cells were seeded at 9,000 cells/well and BEAS-2B cells at 10,000 cells/well onto a 96-well plate. After treatment time, MTT solution was added at a final concentration of 0.5 mg/mL and incubated for 1 h at 37°C and 5% (vol/vol) CO_2_. The MTT solution was completely removed, and to dissolve the formed formazan, 70 µL dimethyl sulfoxide was added and incubated for 45 min at 37°C at a constant shaking rate of 120 rpm. Finally, absorbance was measured at wavelengths 570 and 630 nm using a Synergy H1 Multi-Mode Reader (BioTek, USA) (46).

### Cell death assay

Cell death was determined by staining the cells with propidium iodide (PI) and Hoechst 33258. A549 and BEAS-2B cells were seeded at 20,000 cells/well onto 48-well plates and infected with influenza virus (H1N1/PR8), HAdV-C5 virus, or treated with RSL3 as described above. At the indicated times post-infection, PI (Cat. #V13242; Fisher Scientific, Landsmeer, the Netherlands) and Hoechst 33258 (Cat. #H1398; Invitrogen, the Netherlands) were added to each well at a final concentration of 1 µM and 1 µg/mL, respectively. Cells were incubated at 37°C and 5% (vol/vol) CO_2_ for 10 min and then imaged using a Nikon Inverted Research Fluorescence Microscope ECLIPSE Ti2-E at a nominal magnification of 20×. The number of PI^+^ and Hoechst^+^ cells was quantified using Fiji (47). The average number of Hoechst^+^ cells in the control group was calculated ($\bar{\left({Hoechst}_{Control}^{+}\right)}$), and, for each image, the percentage of cell death relative to control was calculated in the following way:


$$\frac{\left(\bar{{Hoechst}_{Control}^{+}}-{Hoechst}_{Treatment}^{+}\right)+PI_{Treatment}^{+}}{{Hoechst}_{Control}^{+}}$$

Ten images per condition were analyzed.

### Lipid peroxidation

Lipid peroxidation was determined using BODIPY 581/591 C11 Lipid Peroxidation Sensor (Cat. #D3861, Fisher Scientific, Landsmeer, the Netherlands) (46, 48, 49). A549 and BEAS-2B cells were seeded at 20,000 cells/well onto 48-well plates and infected with IAV (H1N1/PR8) or HAdV-C5 or treated with RSL3 as described above. RSL3-treated samples served as a positive control and were analyzed only after 24-h treatment, which was performed concurrently with both H1N1 and HAdV-C5 24 samples at 24 hpi; therefore, the RSL3 data presented are the same for both viruses (Fig. 3 and 6). At the indicated times post-infection, BODIPY 581/591 C11 and Hoechst 33258 dye were added to each well at a final concentration of 1.5 µM and 1 µg/mL, respectively. Cells were incubated at 37°C and 5% (vol/vol) CO_2_ for 15 min and then imaged using a Nikon Inverted Research Fluorescence Microscope ECLIPSE Ti2-E at a nominal magnification of 20×. Ten images per condition per biological replicate were taken and analyzed using Fiji (47).

### Malondialdehyde assay

Malondialdehyde was measured as a marker of lipid peroxidation by the thiobarbituric acid reactive substance assay (46, 50). A549 cells were seeded at 275,000 cells/well and BEAS-2B cells at 320,000 cells/well onto a 6-well plate. Infection was done as described above. Fer-1 treatment was added 1 h prior to infection and removed at 0 hpi. RSL3 treatment was added at the same time of infection. At the indicated times post-infection, cells were washed three times with PBS and collected by scraping in ice-cold water. Cells were lysed by sonication and subjected to three freeze-thaw cycles. A volume of 50 μL of cell lysate was mixed with 100 μL of ice-cold 10% trichloroacetic acid (Cat. #1.00807, Merck) and incubated for 15 min on ice. Samples were centrifuged for 15 min at 5,000× *g* at 4°C, and 100 µL of supernatant was mixed with an equal volume of 0.75% 2-thiobarbituric acid (Cat. #T550, Merck). An MDA (1,1,3,3-tetramethoxypropane, Cat. #108383, Merck) standard curve (0–500 μM) was prepared the same way. Samples and standards were immediately boiled at 95°C for 15 min, then allowed to cool to RT. Fluorescence was measured at λ_Ex_/λ_Em_ = 533/559 nm using a BioRad CFX96 Real-Time PCR System. Total protein concentration of samples was measured using the Pierce BCA Protein Assay Kit (Cat. #23335, Thermo Scientific). MDA concentrations in samples were calculated from the MDA standard curve and normalized by their respective total protein concentration.

### Western blotting

A549 cells were seeded at 275,000 cells/well and BEAS-2B cells at 320,000 cells/well onto a 6-well plate. Infection was done as described above. Samples were collected at the indicated times post-infection in lysis buffer (0.25 M D-Mannitol, 0.05 M Tris-Base, 1 mM EDTA, 1 mM EGTA [pH 7.8], and a mixture of protease inhibitors: 1 mM Na_3_VO_4_, 1 mM NaF, 10 μg/mL leupeptin, 10 μg/mL pepstatin A, 10 μg/mL apoprotein, and 1 mM DTT). Total protein was quantified using the Pierce BCA Protein Assay Kit (Cat. #23335, Thermo Scientific). Equal amounts of protein were loaded and separated by 12% SDS-polyacrylamide gel electrophoresis, transferred onto nitrocellulose membranes, and blocked with 5% milk. Subsequently, membranes were incubated overnight at 4°C with primary antibodies against ferritin light chain (1:1,000, Cat. #ab69090, Abcam) and GAPDH (1:3,000, Cat. # sc-47724, Santa Cruz Technology) in 1% milk. Membranes were washed thoroughly and incubated with horseradish peroxidase-conjugated secondary antibodies (anti-rabbit IgG, 1:3,000; anti-mouse IgG, 1:3,000, respectively) at RT for 1 h. Antibody-labeled proteins were detected using a Western Lightning ECL-plus Chemiluminescent Substrate detection kit (Cat. #50-904-9326, Fisher Scientific). Band intensity was quantified using Image Lab Software version 6.1 (https://www.bio-rad.com/en-nl/product/image-lab-software?ID=KRE6P5E8Z). Expression of ferritin was normalized to GAPDH.

### Mitochondrial staining and morphology analysis

A549 and BEAS-2B cells were seeded at 40,000 cells/well onto glass coverslips, and hAECs were seeded at 65,000 cells/well onto coated coverslips (fibronectin [10 μg/mL], bovine type I collagen [30 μg/mL], and bovine serum albumin [10 μg/mL] in PBS [40]) in a 24-well plate, followed by the virus infections as outlined above. Mitochondria were labeled by incubating the cells with 100 nM MitoTracker Red FM (Cat. #M22425, Invitrogen, Oregon City, OR, USA) for 30 min. Following incubation, cells were washed three times with PBS and fixed with 4% PFA solution at RT for 15 min. After washing three more times with PBS, coverslips were mounted on glass slides using mounting media with DAPI (Cat. #ab104139, Abcam). At least 10 images per condition were acquired using a Nikon Inverted Research Fluorescence Microscope ECLIPSE Ti2-E at a nominal magnification of 60× and analyzed using ImageJ software.

Mitochondrial footprint was analyzed using the MiNA plugin (https://github.com/StuartLab/MiNA/blob/master/README.md) for Fiji (47, 51). At least 10 single-cell images were analyzed for each condition. Branch length per mitochondrion, branches per mitochondrion, branch junctions per mitochondrion, and mean form factor were measured from binarized images using the Mitochondria Analyzer plugin (https://github.com/AhsenChaudhry/Mitochondria-Analyzer) (47, 52). Optimal threshold parameters were first determined and maintained for subsequent analysis for all images.

### Statistical analysis

GraphPad Prism software (version 8.4.2, GraphPad Software Inc., La Jolla, CA, USA) was used to create all graphs and analyze data. All data are expressed as mean ± standard deviation (SD) for all experiments repeated as indicated in the figure legends. Analysis was performed using one-way ANOVA followed by Dunnett’s multiple comparisons or by Tukey’s *post hoc* multiple comparisons test. Significance is indicated by *P < 0.05, **P < 0.01, ***P < 0.001, ****P < 0.0001, or otherwise not significant (n.s.).

## RESULTS

### H1N1-induced cell death of epithelial cells leads to increases in lipid peroxidation and ferritin expression

**Fig 1:**
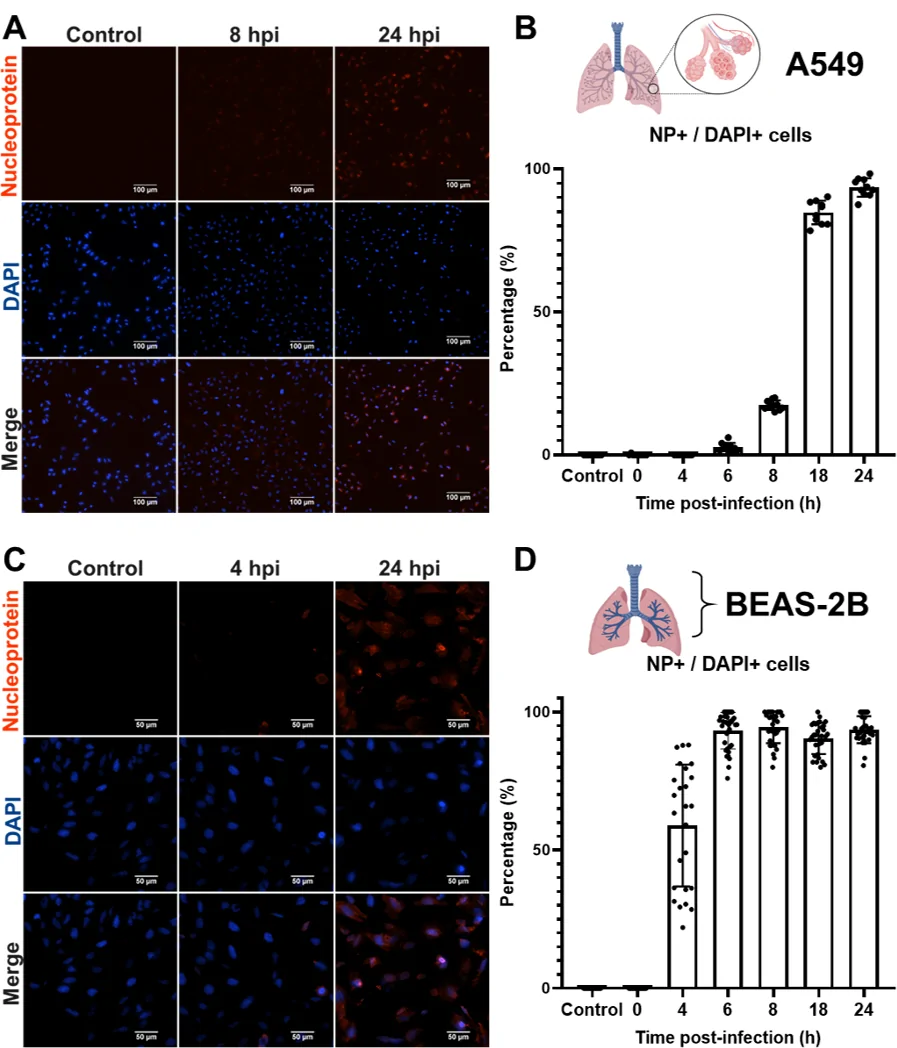
H1N1 replication in lung epithelial cells. Fluorescence images of (**A**) A549 and (**C**) BEAS-2B cells infected with H1N1/PR8 influenza virus at an MOI of 1, fixed and stained at the indicated times post-infection taken at a nominal magnification of 20×. Staining was performed with an anti-influenza nucleoprotein antibody and counterstaining with DAPI. Red, NP-positive cells; blue, nucleus (scale bar = 100 µm for A549 cells and 50 µm for BEAS-2B cells). Quantitative analysis of NP-positive and DAPI-positive (**B**) A549 and (**D**) BEAS-2B cells was performed on at least five images per condition per biological replicate using ImageJ. Results are shown as a percentage of NP^+^/DAPI^+^ cells (mean ± SD).

It was previously demonstrated that influenza virus induces ferroptosis in A549 cells (17, 19). We evaluated whether the H1N1/PR8 strain causes ferroptosis in both alveolar (A549) and bronchial (BEAS-2B) epithelial cells. We first assessed infection dynamics by infecting cells with H1N1/PR8 at a multiplicity of infection of 1 and monitored NP expression at 0, 4, 6, 8, 18, and 24 h post-infection by immunostaining (Fig. S1). In A549 cells, NP^+^ cells reached 84.7% by 18 hpi and 93.5% by 24 hpi (Fig. 1A and B). In contrast, BEAS-2B cells showed faster NP production, with 58.9% cells already being NP^+^ at 4 hpi and peak infection levels (>90%) by 6 hpi (Fig. 1C and D).

**Fig 2:**
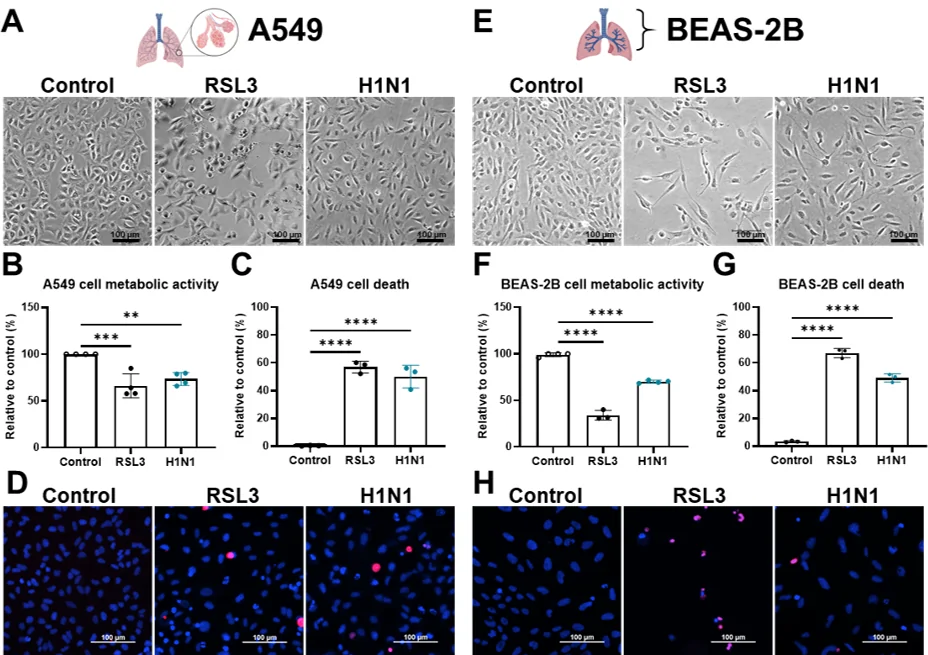
H1N1 causes cell death in lung epithelial cells. Representative brightfield microscope images of (**A**) A549 and (**E**) BEAS-2B cells infected with H1N1/PR8 influenza virus at an MOI of 1 at 24 hpi or treated with RSL3 (A549, 10 µM; BEAS-2B, 250 nM) (scale bar = 100 µm). MTT assay as a measurement of metabolic activity of viable (**B**) A549 and (**F**) BEAS-2B cells. Cell metabolic activity of the control group was defined as 100% activity. Each dot represents a biological replicate with at least six technical replicates per condition. Results are shown as a percentage relative to the control (mean ± SD). Cell death quantification in (**C**) A549 and (**G**) BEAS-2B cells by PI and Hoechst double staining and representative images of (**D**) A549 and (**H**) BEAS-2B cells taken at a nominal magnification of 20×. The number of dead cells was quantified according to the equation stated in Materials and Methods. In short, the total number of dead cells is calculated as the sum of PI^+^ cells and the difference between the average number of DAPI^+^ cells in the control and the number of DAPI^+^ cells in the treatment, divided by the average number of DAPI^+^ cells in the control. Red, PI-positive, dead cells; blue, nucleus (scale bar = 100 µm). Each dot represents the average of 10 images per condition. Results are shown as a percentage of dead cells relative to the number of nuclei present in the control group (mean ± SD). Statistical analysis was performed using one-way ANOVA followed by Dunnett’s multiple comparisons test. **P < 0.01, ***P < 0.001, and ****P < 0.0001. Results are of at least three biological replicates.

Since both cell lines showed maximal infection by 24 hpi, we continued to use this time point for subsequent experiments, with RSL3, a known ferroptosis inducer (53, 54), serving as a positive control. In conjunction with increased NP production, morphological changes and a reduction in cell number were observed in H1N1-infected and RSL3-treated cultures in both A549 (Fig. 2A) and BEAS-2B (Fig. 2E) cells. To investigate whether virus infection leads to changes in metabolic activity, we performed MTT assays, which showed a significant reduction in cell metabolic activity in H1N1-infected A549 (73.48% ± 6.83%) (Fig. 2B) and BEAS-2B (70.03% ± 1.62%) cells (Fig. 2F) compared to their respective controls. Given that the MTT assay measures cell metabolic activity and only indirectly provides insight into cell viability, we sought to quantify cell death by using PI and Hoechst 33258 staining. These results confirmed significant cell death in both A549 (50.1% ± 8.19%) (Fig. 2C and D) and BEAS-2B (49.1% ± 2.94%) (Fig. 2G and H) cells following H1N1 infection, which correlates with our findings in the MTT assay. For comparison, RSL3 treatment induced cell death levels of 56.95% ± 4.21% in A549 and 66.98% ± 3.32% in BEAS-2B cells.

**Fig 3:**
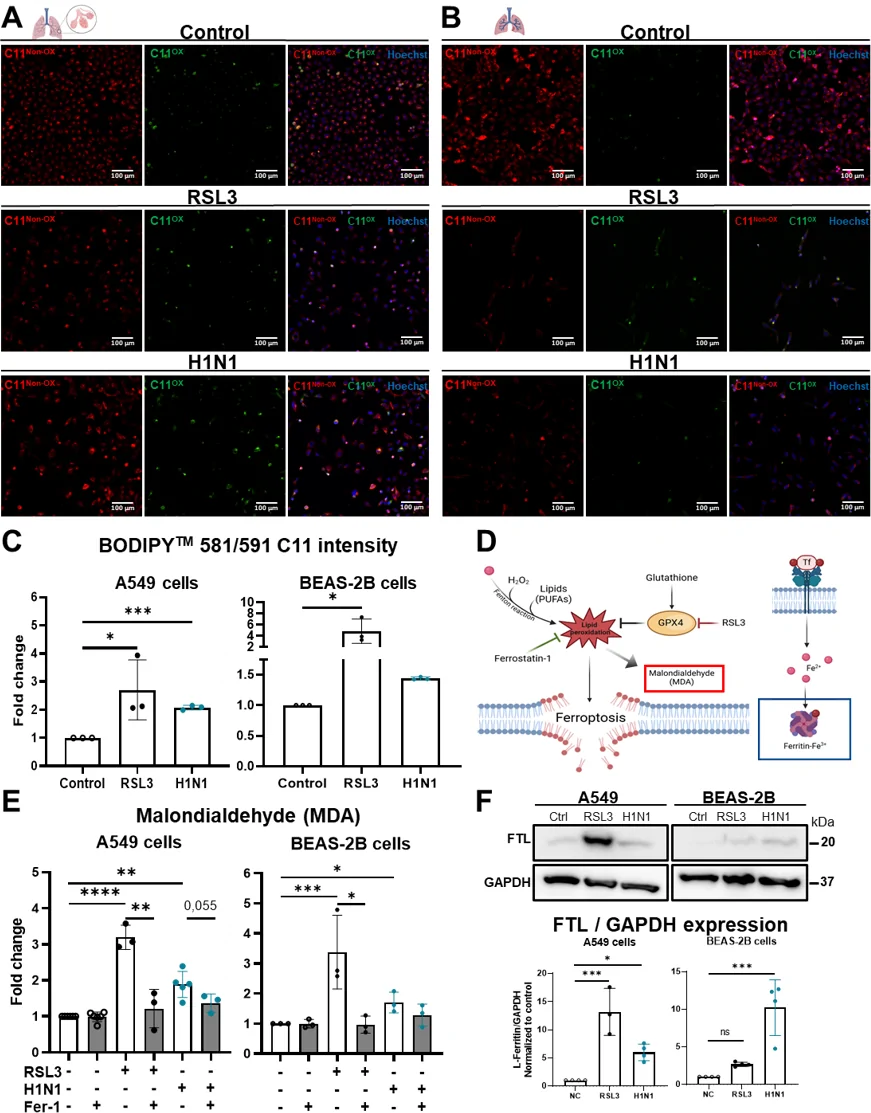
Influenza virus H1N1 causes increased levels of lipid peroxidation and FTL expression in A549 and BEAS-2B cells. Fluorescence images taken at a nominal magnification of 20× of (**A**) A549 and (**B**) BEAS-2B cells treated with RSL3 (A549, 10 µM; BEAS-2B, 250 nM) or infected with H1N1/PR8 influenza virus at an MOI of 1 stained with BODIPY 581/591 C11 at 24 hpi and counterstained with Hoechst 33258. Red, non-oxidized lipids (C11^Non-Ox^); green, oxidized lipids (C11^Ox^); and blue, nucleus (scale bar = 100 µm). Quantitative analysis of the mean integrated fluorescence intensity of C11^Ox^ /C11^Non-Ox^ ratio was performed for both (**C**) A549 and BEAS-2B cells (mean ± SD). Each dot represents the quantification of one biological replicate. At least 10 images per condition per biological replicate were analyzed. C11^Ox^ /C11^Non-Ox^ ratio of the control was defined as 100%. (**D**) Schematic representation of iron participation in the Fenton reaction leading to oxidation of PUFAs, the production of malondialdehyde, and representation of iron transport into the cell by transferrin receptor (Tf), as well as the subsequent storage of intracellular iron in ferritin. The figure panel was created using BioRender (Academic Individual Subscription held by K Rafie). (**E**) MDA levels of A549 and BEAS-2B cell lysates collected at 24 hpi with influenza H1N1/PR8 and treatment with RSL3 (A549, 10 µM; BEAS-2B, 250 nM) and pre-treatment with Fer-1 (5 µM). MDA concentrations were normalized to the corresponding protein concentration and are presented as fold changes to the control. (**F**) Representative blot showing the relative expression of FTL in A549 and BEAS-2B cells infected with influenza H1N1/PR8 at an MOI of 1 or treated with RSL3 (A549, 30 µM; BEAS-2B, 300 nM). Cell lysates were harvested at 24 hpi for Western blot analysis. Statistical analysis was performed using one-way ANOVA followed by Dunnett’s multiple comparisons test. *P < 0.05, **P < 0.01, ***P < 0.001, and n.s., not significant. Data are represented as mean ± SD of at least three independent biological replicates.

A central feature of ferroptosis is the excessive and uncontrolled peroxidation of polyunsaturated fatty acids (PUFAs) present in cell membranes, a process that precedes and contributes to cell death (55, 56). To assess this in our model, we measured lipid peroxidation in H1N1-infected cells at 24 hpi by staining A549 (Fig. 3A) and BEAS-2B (Fig. 3B) cells with the fluorescent probe BODIPY 581/591 C11. Oxidation of the polyunsaturated butadienyl portion of the dye causes a fluorescence emission shift from red (~590 nm) to green (~510), allowing for the quantification of oxidized lipids to non-oxidized lipids. The ratio of green to red fluorescence intensity then allows for the quantification of lipid peroxidation (Fig. 3C).

In A549 cells, H1N1 infection increased the ratio of green to red signal (2.1 ± 0.1-fold) compared to the control group. As expected, the RSL3 treatment caused a more pronounced increase (2.7 ± 1.1-fold) in lipid peroxidation (Fig. 3C, left). Similarly, in BEAS-2B cells, H1N1 infection increased the ratio of green to red signal (1.4 ± 0.1-fold) compared to the control, while RSL3 treatment caused a marked increase (4.8 ± 2.2-fold) (Fig. 3C, right). To confirm whether the increased lipid peroxidation may be linked to ferroptosis, the ferroptosis inhibitor ferrostatin-1 was used to pre-treat cells for 1 h prior to infection or RSL3 treatment followed by the determination of malondialdehyde as a measure of lipid peroxidation (57) (Fig. 3D). In A549 cells, MDA levels significantly increased in H1N1-infected (1.9 ± 0.4-fold) and RSL3-treated groups (3.2 ± 0.3-fold) compared to the control condition (Fig. 3E, left). In BEAS-2B cells, RSL3 treatment induced a similar increase in MDA levels (3.4 ± 1.2-fold), and H1N1 infection caused a lower but significant increase (1.7 ± 0.3-fold) (Fig. 3E, right). As expected, Fer-1 pre-treatment prevented the increase of MDA induced by RSL3 in both cell lines (Fig. 3E). Infected BEAS-2B and A549 cells pre-treated with Fer-1 also showed a lower level of MDA (1.3 ± 0.4-fold and 1.4 ± 0.3-fold, respectively) compared to untreated infected cells (1.7 ± 0.3-fold and 1.9 ± 0.4-fold, respectively).

The excess oxidation of PUFAs is driven by labile, ferrous iron (Fe^2+^) participating in the Fenton reaction, indicating that processes involved in the import, export, and storage of iron may be tightly regulated to prevent lipid peroxidation (55). Ferritin, the major iron storage protein in cells (Fig. 3D), is known to play an important role in ferroptosis and in the replication of H1N1 (19); therefore, we next determined whether ferritin expression levels were altered in H1N1-infected cells. In A549, ferritin expression levels significantly increased (6.0 ± 1.5-fold) in H1N1-infected cells (Fig. 3F), in a similar trend to the RSL3-treated group (13.2 ± 4.2-fold) relative to the control. In BEAS-2B cells infected with H1N1, ferritin expression levels significantly increased (10.2 ± 3.7-fold) compared to the control. This increase in ferritin expression levels of infected cells confirms a disruption of intracellular iron homeostasis and suggests a compensating mechanism in response to an increase in intracellular labile iron. Consistent with previous reports (17, 19), our results indicate that H1N1 influenza virus infection causes ferroptosis features, as evidenced by increased lipid peroxidation, MDA levels, and ferritin protein expression in A549 and BEAS-2B cells.

### Human adenovirus type C5 causes cell death at longer times of incubation

Next, we investigated whether ferroptosis features are also triggered by human adenovirus type C5. To assess infection dynamics and capture potential early infection events, cells were infected with HAdV-C5 at an MOI of 1, and the expression of hexon, a major structural protein found in the capsid of HAdVs, was monitored as a marker of infection. Since HAdV structural proteins can take over 36 hpi to become detectable (58), we stained for hexon protein at 24, 48, 72, and 96 hpi (Fig. S2).

**Fig 4:**
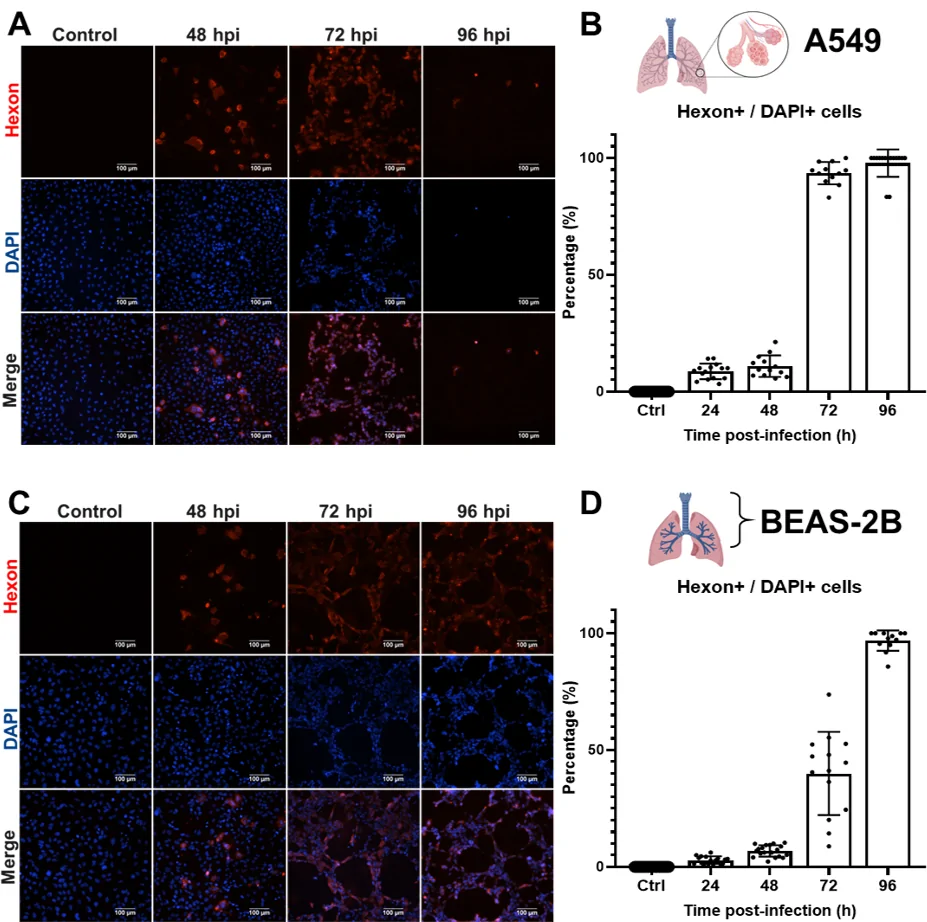
HAdV-C5 replication rate in lung epithelial cells. Fluorescence images taken at a nominal magnification of 20× of (**A**) A549 and (**C**) BEAS-2B cells infected with HAdV-C5 virus at an MOI of 1 fixed and stained at 48, 72, and 96 hpi with anti-hexon antibody and counterstained with DAPI. Red, hexon-positive cells; blue, nucleus (scale bar = 100 µm). Quantitative analysis of hexon-positive and DAPI-positive (**B**) A549 and (**D**) BEAS-2B cells was performed on at least 10 images per condition using ImageJ. Results are shown as the percentage of Hexon^+^/DAPI^+^ cells (mean ± SD).

In A549 cells, it took over 48 hpi for most cells to become hexon^+^, with nearly all cells (93.48% ± 4.75%) being hexon^+^ by 72 hpi, whereas at 96 hpi only a few nuclei were visible, indicating a substantial cytopathic effect took place (Fig. 4A). In contrast, in BEAS-2B cells, it took over 72 hpi for the majority of cells (96.81% ± 4.36%) to become hexon^+^, indicating a slower infection rate in bronchial epithelial cells (Fig. 4D).

**Fig 5:**
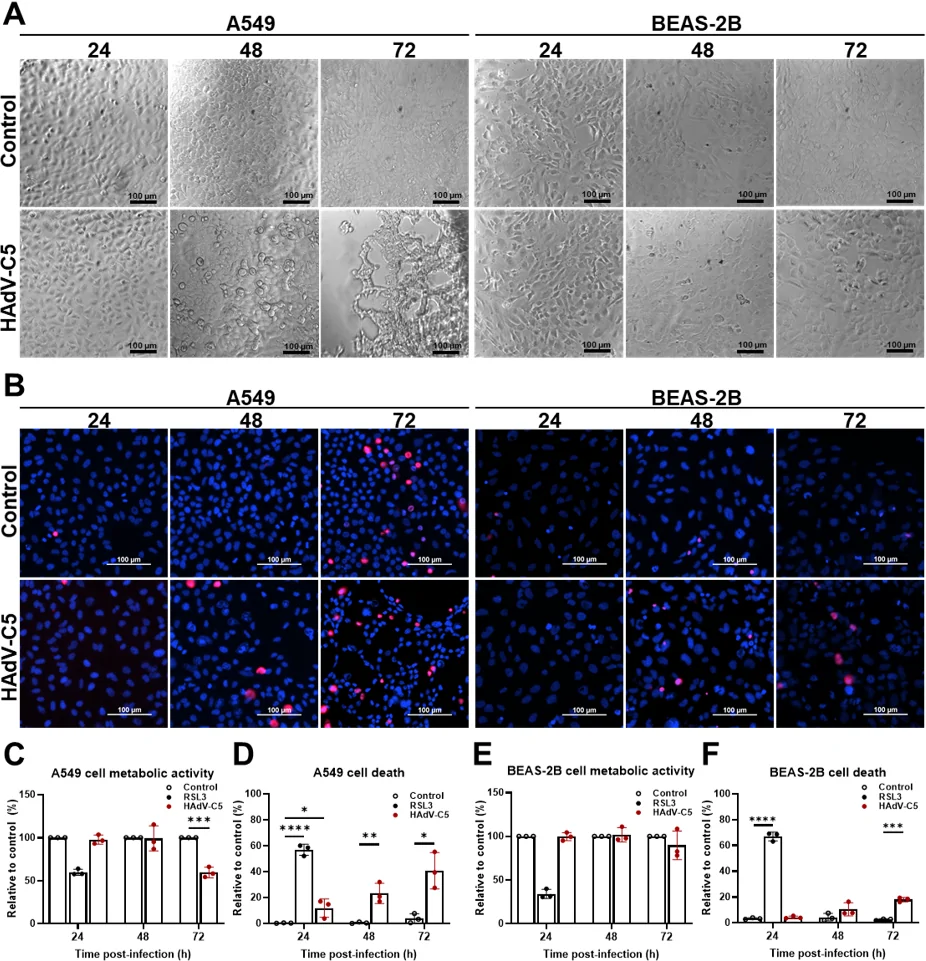
HAdV-C5-induced cell death has an earlier onset in A549 cells compared to BEAS-2B cells. (**A**) Representative brightfield microscope images of A549 and BEAS-2B cells infected with HAdV-C5 virus at an MOI of 1 at 24, 48, and 72 hpi (scale bar = 100 µm). (**B**) Representative fluorescence images of PI and Hoechst double staining taken at a nominal magnification of 20×. Red, PI-positive, dead cells; blue, nucleus (scale bar = 100 µm). MTT assay as a measurement of metabolic activity of viable (**C**) A549 and (**E**) BEAS-2B cells. Cell metabolic activity of the control group was defined as 100% activity. Each dot represents a biological replicate with at least six technical replicates per condition. Results are shown as a percentage relative to the control (mean ± SD). Cell death of (**D**) A549 and (**F**) BEAS-2B as measured by PI staining. RSL3 at 24 h was introduced as a single-time-point positive control. The number of dead cells was quantified according to the equation stated in Materials and Methods. In short, the total number of dead cells is calculated as the sum of PI^+^ cells and the difference between the average number of DAPI^+^ cells in the control and the number of DAPI^+^ cells in the treatment, divided by the average number of DAPI^+^ cells in the control. Each dot represents the average of 10 images per condition (mean ± SD). Statistical analysis was performed using one-way ANOVA followed by Dunnett’s multiple comparisons test. *P < 0.05, **P < 0.01, ***P < 0.001, ****P < 0.0001, and ns: non-significant. Results are of at least three biological replicates.

At 48 hpi, morphological changes consistent with the cytopathic effect of HAdVs, such as cell rounding and swelling, were observed in A549 (Fig. 5A, left). In contrast, comparable morphological changes were observed in BEAS-2B cells only at 72 hpi (Fig. 5A, right). PI staining revealed an increasing number of PI^+^ cells over time in A549, but not in BEAS-2B cells (Fig. 5B). Consistent with these findings, cell metabolic activity in A549 cells was significantly reduced (59.6% ± 6.1%) by 72 hpi (Fig. 5C). Quantification of PI^+^ cells showed a significant increase in cell death at 48 hpi (23.30% ± 7.83%), with a further increase at 72 hpi (40.77% ± 13.94%) (Fig. 5D). In BEAS-2B cells, metabolic activity remained unaffected up to 72 hpi, and PI quantification showed only a small increase in cell death at 72 hpi (18.0% ± 1.7%) (Fig. 5E and F). Importantly, cell death caused by HAdV-C5 at 72 hpi was not prevented by apoptosis inhibitors Z-DEVD-FMK, Z-VAD-FMK, and Q-VD-Oph in A549 and BEAS-2B cells (Fig. S4), suggesting that although apoptosis is known to be an RCD mechanism induced by HAdV-C5 infection (2, 25), other forms of RCD, such as ferroptosis, are involved.

**Fig 6:**
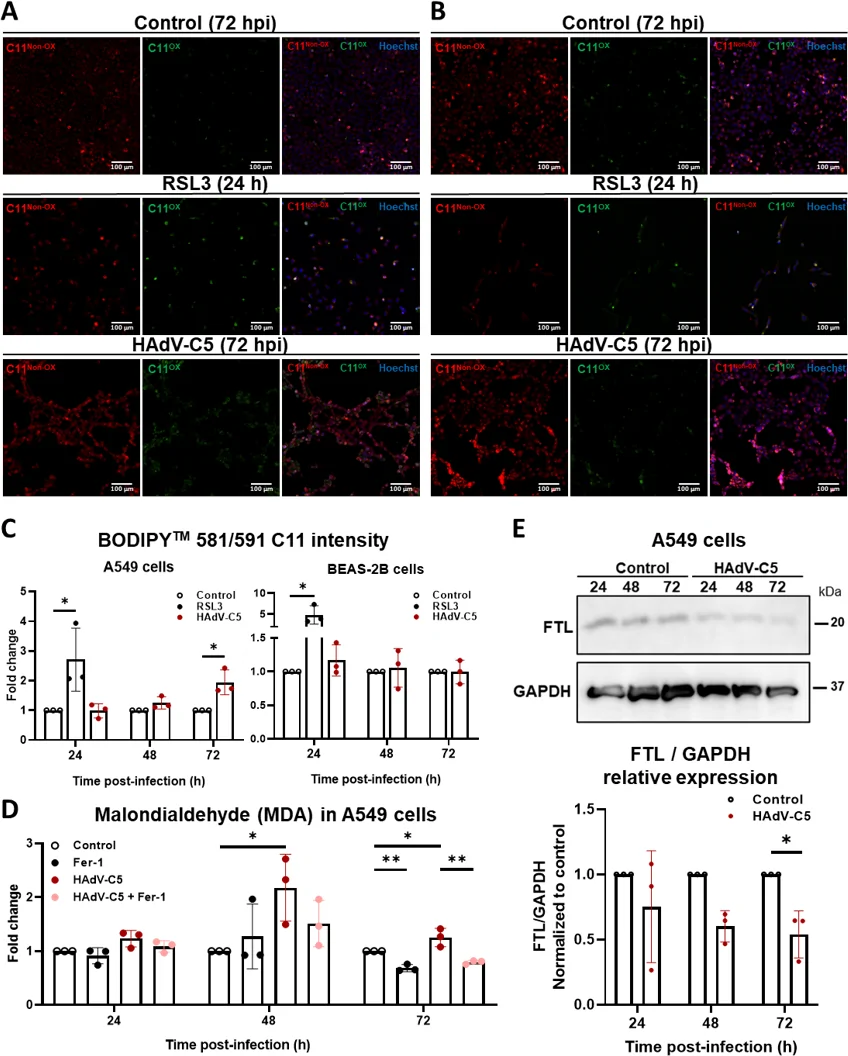
HAdV-C5-induced lipid peroxidation in A549 cells in a time-dependent manner. Representative fluorescence images taken at a nominal magnification of 20× of (**A**) A549 and (**B**) BEAS-2B cells treated with RSL3 (A549, 10 µM; BEAS-2B, 250 nM) for 24 h or infected with HAdV-C5 at an MOI of 1 stained with BODIPY 581/591 C11 at 72 hpi and counterstained with Hoechst 33258. RSL3 positive control treatment was performed concurrently with the 24 hpi H1N1 and HAdV-C5 experiments; therefore, the same data as that presented in the H1N1 panel are reproduced here for comparison (as in Fig. 3). Red, non-oxidized lipids (C11^Non-Ox^); green, oxidized lipids (C11^Ox^); blue, nucleus (scale bar = 100 µm). (**C**) Quantitative analysis of mean integrated fluorescence intensity of C11^Ox^/C11^Non-Ox^ ratio was measured for both A549 and BEAS-2B cells (mean ± SD) at 24, 48, and 72 hpi. Each dot represents the quantification of one biological replicate. At least 10 images per condition per biological replicate were analyzed. C11^Ox^/C11^Non-Ox^ ratio of the control was defined as 100%. Statistical analysis was performed using one-way ANOVA followed by Dunnett’s comparisons test. (**D**) MDA levels of A549 cell lysates collected at 24, 48, and 72 hpi with HAdV-C5 and treatment with RSL3 (10 µM) and pre-treatment with Fer-1 (5 µM). MDA concentration was normalized to the corresponding protein concentration and is presented as fold changes compared to the control. (**E**) Representative blot showing the relative expression of FTL in A549 cells infected with HAdV-C5 at an MOI of 1 or treated with RSL3 (30 µM). Cell lysates were harvested at 24, 48, and 72 hpi for Western blot analysis. Data are represented as mean ± SD of at least three independent biological experiments with three technical replicates. Statistical analysis was performed using one-way ANOVA followed by Tukey’s *post hoc* multiple comparisons test. *P < 0.05 and **P < 0.01.

Next, lipid peroxidation was measured by staining with BODIPY 581/591 C11 following infection with HAdV-C5 at 24, 48, and 72 hpi in A549 (Fig. 6A) and BEAS-2B (Fig. 6B) cells. RSL3 treatment at 24 h served as a positive control (same as for H1N1, as the RSL3 and HAdV-C5 samples were prepared in the same experiment). Quantification of green-to-red fluorescence intensity ratios showed a time-dependent increase of oxidized lipids in A549 cells, with a significant change at 72 hpi (1.9 ± 0.4-fold) compared with the respective 72 h control. In contrast, no change in the ratio of oxidized to non-oxidized lipids was observed in BEAS-2B cells at any of the time points measured (Fig. 6D).

Based on these findings, which indicate a higher susceptibility of A549 cells to HAdV-C5 infection, and the absence of observable changes in cell viability or lipid peroxidation in BEAS-2B cells, follow-up experiments were performed only on A549 cells. To further confirm the increase in lipid peroxidation, MDA levels were measured and assessed for sensitivity to the ferroptosis inhibitor Fer-1. MDA levels were significantly increased as early as 48 hpi (2.2 ± 0.6-fold) and remained significantly higher at 72 hpi (1.3 ± 0.2-fold) compared with their respective controls (Fig. 6D). Pre-treatment with Fer-1 significantly reduced the amount of MDA compared to non-Fer-1 pre-treated samples at 72 hpi in both control (0.7 ± 0.06-fold vs 1-fold) and HAdV-C5-infected (0.8 ± 0.03-fold vs 1.3 ± 0.2-fold) cells (19, 36). Finally, contrary to H1N1 infections of A549, the expression of FTL was found to be lower compared to control conditions; however, it did not significantly change between sampled time points, only showing a significant decrease at 72 h (Fig. 6E).

Overall, our data indicate that HAdV-C5 induces a marked increase in lipid peroxidation at later times of infection, which correlates with observable changes in cell morphology and hexon expression. However, the increase in MDA was only partially reduced by Fer-1, and no changes in FTL expression were detected, suggesting an iron-independent increase in lipid peroxidation.

### H1N1 and HAdV-C5 infections cause alterations to the mitochondrial network in bronchial epithelial cells

Mitochondria regulate lipid metabolism, iron transport, and reactive oxygen species (ROS) production and thus play an active role in triggering ferroptotic cell death (10). When exposed to ferroptosis inducers, such as RSL3, the mitochondrial outer membrane ruptures, causing the mitochondria to appear compacted and fragmented (4, 10). Viruses are known to manipulate mitochondrial dynamics and morphology to benefit their own replication and survival (59, 60). Therefore, we aimed to determine the effect of H1N1 and HAdV-C5 infection on the mitochondrial network and changes in morphology.

**Fig 7:**
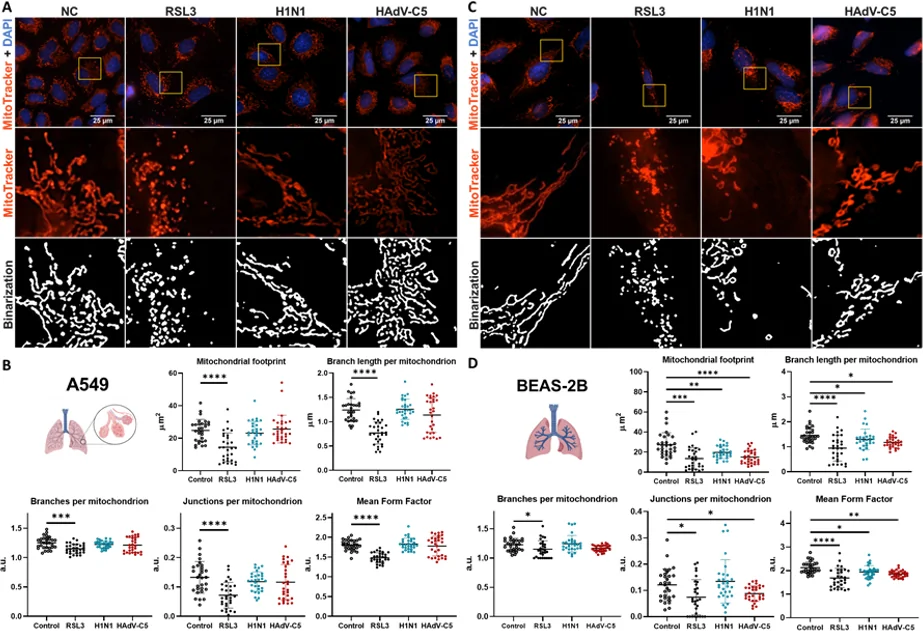
Influenza H1N1 and HAdV-C5 infections cause alterations in mitochondrial morphology in BEAS-2B but not A549 cells. MitoTracker Red FM fluorescence images of (**A**) A549 and (**C**) BEAS-2B cells at a nominal magnification of 60× after treatment with RSL3 (A549, 10 µM; BEAS-2B, 250 nM) for 24 h or infection with influenza H1N1/PR8 or HAdV-C5 at an MOI of 1 at 24 hpi; Red, mitochondria; blue, nucleus (scale bar = 25 µm). Enlarged images of the boxed area are shown for MitoTracker and after binarization using the Mitochondria Analyzer plugin (47, 52). Parameters of mitochondrial network analysis for (**B**) A549 and (**D**) BEAS-2B cells. Mitochondrial footprint of at least 10 single cells was analyzed for each condition (mean ± SD). Branch length per mitochondrion, branches per mitochondrion, junctions per mitochondrion, and mean form factor of at least 10 whole images per condition were determined (mean ± SD). Statistical analysis was performed using one-way ANOVA followed by Dunnett’s multiple comparisons test. *P < 0.05, **P < 0.01, ***P < 0.001, and ****P < 0.0001. Results are of at least three biological replicates.

Morphological changes to mitochondria have been observed in H1N1-infected A549 cells at 24 hpi (18, 61). To recapitulate this phenotype, we stained cells with MitoTracker Red FM and visualized the mitochondrial network with fluorescence microscopy images, which were binarized using the Mitochondria Analyzer plugin (47, 52) for the quantification of mitochondrial morphology and the MiNA plugin (47, 51) for the quantification of the footprint (area) occupied by mitochondria. We observed that mitochondria formed long, tubular-like structures in control A549 cells (Fig. 7A). In comparison, mitochondria in the RSL3-treated groups appeared fragmented and dot-like (Fig. 7A), observations confirmed by the significant decrease in mitochondrial footprint (14.4 ± 8.9 µm^2^ vs 24.8 ± 6.8 µm^2^), branch length per mitochondrion (0.76 ± 0.2 µm vs 1.2 ± 0.2 µm), number of branches per mitochondrion (1.2 a.u. ± 0.1 vs 1.3 ± 0.1 a.u.), junctions per mitochondrion (0.07 ± 0.04 a.u. vs 0.1 ± 0.06 a.u.), and mean form factor (1.5 ± 0.1 a.u. vs 1.8 ±0.1 a.u.), indicating less and smaller, rounder mitochondria (Fig. 7B). In contrast, only small changes in mitochondrial network structure could be seen in H1N1 and HAdV-C5-infected A549 cells, with more doughnut-shaped mitochondria appearing compared to the control. However, quantification confirmed no significant changes in mitochondrial footprint and morphology parameters, such as branch length and junctions per mitochondrion and mean form factor, caused by the viruses at 24 hpi (Fig. 7B).

Interestingly, BEAS-2B cells infected with H1N1 had mitochondria that appeared shorter and doughnut-shaped (Fig. 7C). Indeed, quantification of mitochondrial footprint and morphology parameters showed a significant decrease in mitochondrial footprint (20 ± 6 µm^2^ vs 27 ± 12 µm^2^), branch length per mitochondrion (1.3 ± 0.4 µm vs 1.5 ± 0.3 µm), and mean form factor (1.9 ± 0.3 a.u. vs 2.1 ± 0.3 a.u.), indicating rounder mitochondria with shorter branches compared to controls (Fig. 7D). Similarly, BEAS-2B cells infected with HAdV-C5 exhibited shorter mitochondrial branches (Fig. 7C), which was reflected by the significant reduction in mitochondrial footprint (15 ± 7 µm^2^ vs 27 ± 12 µm^2^), branch length per mitochondrion (1.2 ± 0.2 µm vs 1.5 ± 0.3 µm), junctions per mitochondrion (0.09 ± 0.03 a.u. vs 0.12 ± 0.06 a.u.), and mean form factor (1.9 ± 0.1 a.u. vs 2.1 ± 0.3 a.u.) compared to controls (Fig. 7D). Profound changes could be seen in the RSL3-treated cells that appeared fragmented and dot-like. These observations were confirmed by the significant reduction in mitochondrial footprint (13.6 ± 10.7 µm^2^ vs 27 ± 12 µm^2^), branch length per mitochondrion (0.95 ± 0.5 µm vs 1.5 ± 0.3 µm), branches per mitochondrion (1.15 ± 0.1 a.u. vs 1.23 ± 0.1 a.u.), junctions per mitochondrion (0.08 ± 0.07 a.u. vs 0.12 ± 0.06 a.u.), and mean form factor (1.7 ± 0.4 a.u. vs 2.1 ± 0.3 a.u.) compared to controls (Fig. 7D).

Since the changes caused by HAdV-C5 infection were observed only at later times of infection, we stained the mitochondria of A549-infected cells at 48, 60, and 72 hpi. Images showed a clear reduction in tubular-like structures and an increase in fragmented, dot-like mitochondria that could be observed in the HAdV-C5-infected cells at 48 hpi compared to the control group. By 72 hpi, only a few mitochondria could be observed in the infected cells, all of which appeared dot-like and compacted compared to the control cells at the same time point. The progressive deterioration in mitochondrial morphology (Fig. S3) coincided with time points at which we observed increased lipid peroxidation and cell death. However, accurate quantification of mitochondrial footprint and morphology parameters was not possible due to the mitochondrial signal spanning over multiple imaging layers (52).

Overall, our results indicate that H1N1 and HAdV-C5 infection decrease mitochondrial footprint in BEAS-2B cells and cause detrimental changes to mitochondrial morphology. The level of mitochondrial alterations in these cells was comparable to the damage caused by RSL3.

### H1N1 and HAdV-C5 infections cause alterations to the mitochondrial network in primary human airway epithelial cells

**Fig 8:**
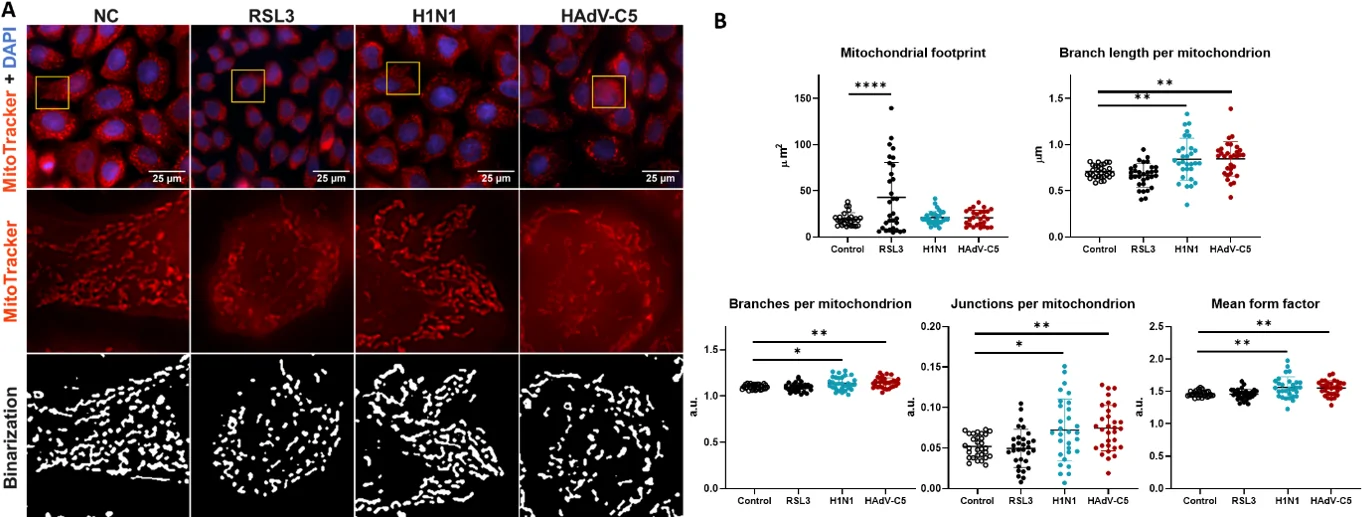
Influenza H1N1 and HAdV-C5 infections cause alterations in mitochondrial morphology in primary human airway epithelial cells. (**A**) MitoTracker Red FM fluorescence images of hAECs at a nominal magnification of 60× after treatment with RSL3 (1 µM) for 24 h or infection with influenza H1N1/PR8 or HAdV-C5 at an MOI of 1 at 24 hpi. Red, mitochondria; blue, nucleus (scale bar = 25 µm). Enlarged images of the boxed area are shown for MitoTracker and after binarization using the Mitochondria Analyzer plugin (43, 48). (**B**) Parameters of mitochondrial network analysis. Mitochondrial footprint of at least 10 single cells was analyzed for each condition (mean ± SD). Branch length per mitochondrion, branches per mitochondrion, junctions per mitochondrion, and mean form factor of at least 10 whole images per condition were determined (mean ± SD). Statistical analysis was performed using one-way ANOVA followed by Dunnett’s multiple comparisons test. *P < 0.05, **P < 0.01, and ****P < 0.0001. Results are of three biological replicates.

To be able to translate our findings to human disease conditions, we infected primary hAECs with H1N1 and HAdV-C5 at an MOI of 1 or treated them with RSL3. At 24 hpi, we stained the cells with MitoTracker Red FM and analyzed mitochondrial morphology. In control cells, we observed that mitochondria formed both tubular and donut-like structures. In contrast, mitochondria in the RSL3-treated groups showed a broader range of morphologies, including shorter, more condensed mitochondria as well as longer and expanded structures (Fig. 8A). Quantitative analysis confirmed a significant increase in mitochondrial footprint in the RSL3-treated cells (19.3 ± 7.2 µm^2^ vs 43.0 ± 37.8 µm^2^) with no changes in the branch length per mitochondrion (0.71 ± 0.07 µm vs 0.67 ± 0.13 µm), number of branches per mitochondrion (1.1 ± 0.03 a.u. vs 1.1 ± 0.04 a.u.), junctions per mitochondrion (0.05 ± 0.01 a.u. vs 0.05 ± 0.02 a.u.), or mean form factor (1.5 ± 0.04 a.u. vs 1.5 ±0.08 a.u.).

In contrast to RSL3 treatment, mitochondrial footprint was not altered in hAECs infected with H1N1 or HAdV-C5 (19.3 ± 7.2 µm^2^ vs 21.1 ± 7.23 µm^2^ and 20.07 ± 8.41 µm^2^, respectively). Interestingly, the mitochondrial network in both H1N1- and HAdV-C5-infected cells appeared more branched and expanded compared to the control group and the findings in cell lines. Indeed, results indicate a significant increase in the branch length per mitochondrion (0.71 ± 0.07 µm vs 0.84 ± 0.22 µm and 0.84 ± 0.18 µm), number of branches per mitochondrion (1.1 ± 0.03 a.u. vs 1.14 ± 0.07 a.u. and 1.14 ± 0.05 a.u.), junctions per mitochondrion (0.05 ± 0.01 a.u. vs 0.07 ± 0.04 a.u. and 0.07 ± 0.03 a.u.), and mean form factor (1.5 ± 0.04 a.u. vs 1.56 ± 0.16 a.u. and 1.55 ± 0.11 a.u.). Taken together, our results demonstrate that H1N1 and HAdV-C5 infections cause remodeling of the mitochondrial network in hAECs toward a more interconnected and branched network, with only a small increase in the mitochondrial footprint.

## DISCUSSION

In this study, we investigated ferroptosis-like hallmarks induced by H1N1 and HAdV-C5 infections in BEAS-2B (bronchial) and A549 (alveolar) epithelial cells. We provide evidence that influenza A virus (H1N1) induces lipid peroxidation and disrupts iron metabolism (Fig. 3), two key hallmarks of ferroptosis, positioning our findings in line with earlier reports (17, 19). Further studies reported a downregulation of FTH1 expression in A549 cells infected with H1N1/PR8 at a low MOI (0.1) (19), whereas our data show an increase in the expression of FTL in both A549 and BEAS-2B cells following infection with H1N1/PR8 at a higher MOI of 1. Proteomics data of normal human bronchial epithelial cells infected with different strains of IAV at an MOI of 2 indicate that the relative expression levels of FTH1 fluctuate in a time-dependent manner (62). Indeed, clinical studies showed, using direct quantification methods of serum ferritin levels, that high ferritin levels are linked to a more severe infection and can be used as a marker for severe complications and poor patient outcomes (63, 64). Furthermore, the upregulation in FTL was comparable to that seen in RSL3-treated groups, suggesting a potential compensatory response, likely resulting from the upregulated expression of transferrin and transferrin receptor that has been previously reported in H1N1-infected A549 cells (17).

We further demonstrate, for the first time, that HAdV-C5 infection also induces lipid peroxidation in A549 cells at later times of infection (48 and 72 hpi) but not at early time points of infection (24 hpi) (Fig. 6) as indicated by the quantification of hexon protein expression (Fig. 4). These findings indicate that lipid peroxidation is a late-stage event in HAdV-C5 infection, rather than an early response. Strikingly, we did not see an increase in lipid peroxidation in BEAS-2B cells within the time frame of our experiments. However, given that HAdV-C5 replication kinetics and onset of cell death in BEAS-2B cells are different compared to A549 cells, it may indicate that cellular pathways may be regulated differently, eliciting an RCD pathway independent of ferroptosis-like hallmarks. Another explanation is that HAdVs elicit different innate and inflammatory responses during infection in different airway epithelial cells (65, 66), some of which may suppress lipid peroxidation cascades.

Interestingly, previous studies, performed in NHI3T3 fibroblasts, have shown that expression of the adenovirus early region 1A (E1A) gene can block the expression of ferritin in response to oxidative stress (29), further showing differential regulation of ferroptosis-like hallmarks in different cell types. We observed a trend toward a decrease in the expression of FTL in A549 cells infected with HAdV-C5. However, whether this trend reflects E1A-mediated suppression of ferritin or the increase in lipid peroxidation observed is unrelated to iron metabolism remains to be clarified.

Beyond lipid peroxidation and ferritin expression, we also explored virus-induced alterations in mitochondrial footprint and morphology. Mitochondria actively contribute to the onset of cysteine-deprivation-induced ferroptosis through glutathione depletion and consequently the generation of lipid peroxides (11). Erastin-induced ferroptosis dramatically changes mitochondrial morphology, causing an increase in mtROS, leading to loss of mitochondrial membrane potential (67). The influenza A virus PB1-F2 protein is known to induce mitophagy by translocating to mitochondria and mediating its degradation by binding to LC3 to promote degradation and elimination of mitochondrial antiviral signaling proteins to impair innate immunity (68–70). Here, we show a decrease in mitochondrial content and morphology in BEAS-2B cells, but not in A549 cells, following H1N1 infection at 24 hpi (Fig. 7). This cell-type-specific effect may be caused by the distinct pattern of sialic acids on bronchial epithelial cells, which promotes H1N1 uptake (14, 71) and results in faster infection in BEAS-2B cells compared to A549 cells (Fig. 1). Furthermore, a study on mitochondrial morphology changes in H1N1-infected A549 cells also found distinct fragmentation patterns under starvation conditions, confirming the findings presented in the present study (61).

Strikingly, the number of studies done on mitochondrial network changes during IAV or HAdV-C5 infections is sparse and limited to the model cell lines also utilized in this study. To the best of our knowledge, this is the first study showing changes in the mitochondrial network in primary hAECs infected with IAV and HAdV-C5, adding an important finding to the respiratory virus research field. It needs to be pointed out that studies on primary airway and nasal epithelial cells have been performed, showing alterations to mitochondrial function (72–74); however, they have not investigated changes in the mitochondrial network structure. Our findings reveal an increase in mitochondrial network parameters (Fig. 8), which is contrary to our findings in model cell lines (Fig. 7). A previous study done by Pila-Castellano et al. (61) reported an elongation of the mitochondrial network, in line with our findings in hAECs. Interestingly, these changes were only seen in infected cells maintained under regular, non-starvation medium conditions, differing from our findings in A549 cells, which were maintained in 1% FBS during the duration of the experiment. Coinciding with these findings, Pila-Castellano et al. (61) also reported a reduction in dynamin-related protein 1 (DRP1) localization to mitochondria, whereas OPA1 localization was increased, which offers a molecular insight into why these changes were observed. Due to the lack of systematic exploration of mitochondrial network morphology during viral infections, we cannot currently suggest a clear underlying mechanism that explains why hAECs, under starvation conditions, show a mitochondrial fusion phenotype and will thus need to be addressed in future studies. Strikingly, treatment of hAECs with RSL3 leads to a marked increase in mitochondrial footprint, whereas the other mitochondrial network parameters were unchanged. This is in line with previously published work on hAECs that showed no measurable changes in mitochondrial volume upon treatment with a higher concentration of RSL3 (10 µM) (75).

Adenoviruses are also known to modulate mitochondrial homeostasis to enhance virus replication (76–78). The viral E1B-19k protein localizes to mitochondria, where it binds to the pro-apoptotic proteins Bax and Bak to prevent apoptosis (77, 79–82). Recently, the viral minor capsid protein VI was shown to localize to mitochondria during late stages of infection and promote, via its membrane-lytic function, the release of mitochondrial DNA and heat shock protein 60 into the cytosol (83), both of which are important for maintaining mitochondrial integrity (84). We observed a decrease in mitochondrial footprint and changes in mitochondrial morphology in BEAS-2B cells infected with HAdV-C5 at early times of infection (24 hpi) that could not be seen in A549 cells at the same time point (Fig. 7). These findings further suggest that adenovirus-induced disruption of mitochondrial homeostasis is both cell type- and time-dependent and is likely also connected to the lytic function of protein VI (83). Indeed, the drastic changes in mitochondrial morphology at later time points post-HAdV-C5 infection prevented reliable quantification of changes in the mitochondrial network, as diffusion of the MitoTracker signal did not allow for accurate determination of the parameters, which is a known limitation of the software as specified by the developer (52).

Recent research shows that mitochondrial integrity is regulated by the localization of cyclic GMP-AMP (cGAMP) synthase (cGAS) to the outer mitochondrial membrane, where it associates with dynamin-related protein 1, one of the known key regulators of mitochondrial fission, and facilitates its oligomerization to control excessive ROS production and limit lipid peroxidation, thereby inhibiting ferroptosis (85). Importantly, cGAS, a cytosolic DNA sensor that, following its binding to DNA, catalyzes the synthesis of second messenger cGAMP to activate stimulator of interferon genes and the downstream production of interferons (86), was found to be significantly increased in cancer cells, including A549 cells (85). The higher cGAS expression in A549 cells may explain why we only observed smaller changes in the mitochondrial network in H1N1- and HAdV-C5-infected cells at 24 hpi. In addition, levels of DRP1 were shown to be differentially altered during IAV infection at early and late stages of infection (61, 87). Interestingly, it was further shown that increasing cyclic adenosine monophosphate (cAMP) levels activates protein kinase A (PKA), which is subsequently translocated to the outer mitochondrial membrane via A-kinase anchoring protein (AKAP), where it can phosphorylate DRP1, thereby inhibiting its function (88). Importantly, it was previously shown that mitochondrial integrity and function are linked to cAMP levels and signaling in lung epithelial cells (35, 89). Indeed, other studies have shown that inhibition of either exchange protein activated by cAMP 1 (EPAC1) or phosphodiesterase 4 reduces IAV replication efficacy, suggesting a potential link between cAMP levels (90, 91), mitochondrial function and morphology, and viral replication. Strikingly, it was shown that HAdV infections can hijack cAMP signaling via E1A-mediated mimicry of AKAP functionality and binding of PKA, suggesting a similar mechanism to that of IAV (92). However, our knowledge on HAdV-induced changes in DRP1 or other mitochondrial integrity regulators is limited. Therefore, future studies should address how viruses alter cAMP-linked signaling and its impact on mitochondrial function.

### Conclusions

In conclusion, we confirmed previous findings regarding the role of ferroptosis during H1N1 infection by demonstrating increased lipid peroxidation, elevated MDA levels, and altered FTL expression in two different lung epithelial cell types. Importantly, we provide novel evidence that HAdV-C5 infection induces lipid peroxidation and MDA accumulation at later stages of infection, a process that is prevented by pre-treatment with the ferroptosis inhibitor Fer-1 only at advanced stages of infection and is not accompanied by significant changes in FTL expression. Additionally, we showed for the first time that both H1N1 and HAdV-C5 infections lead to significant changes in mitochondrial content and morphology in bronchial (BEAS-2B) epithelial cells at early times of infection comparable to mitochondrial changes caused by RSL3. Contrastingly, in alveolar A549 cells, significant mitochondrial changes were observed only at later times of infection with HAdV-C5, whereas H1N1-induced changes recapitulate phenotypes that were seen before (61). In addition, our observations of changes in the mitochondrial network in primary human lung epithelial cells add novel findings to the field of respiratory virus and mitochondrial research, which offer the basis for future research investigating mitochondrial dynamics during viral infections. Taken together, our comparative study of H1N1- and HAdV-C5-induced ferroptosis-like hallmarks offers a more extended and detailed understanding of how lipid peroxidation, iron homeostasis, and mitochondrial morphology are altered. However, whether the cell death caused by them is ferroptosis or a blend of various regulated cell death pathways will require further investigation.

## SUPPLEMENTAL MATERIAL

Supplemental figures spectrum.01957-26-s0001.docx Fig. S1 to S4.

ASM does not own the copyrights to Supplemental Material that may be linked to, or accessed through, an article. The authors have granted ASM a non-exclusive, world-wide license to publish the Supplemental Material files. Please contact the corresponding author directly for reuse.

## References

[1] Rex DAB, Keshava Prasad TS, Kandasamy RK. 2022. Revisiting regulated cell death responses in viral infections. Int J Mol Sci 23:7023. doi:10.3390/ijms23137023 PMC9266763 35806033

[2] Rao L, Debbas M, Sabbatini P, Hockenbery D, Korsmeyer S, White E. 1992. The adenovirus E1A proteins induce apoptosis, which is inhibited by the E1B 19-kDa and Bcl-2 proteins. Proc Natl Acad Sci USA 89:7742–7746. doi:10.1073/pnas.89.16.7742 PMC49787 1457005

[3] Takizawa T, Matsukawa S, Higuchi Y, Nakamura S, Nakanishi Y, Fukuda R. 1993. Induction of programmed cell death (apoptosis) by influenza virus infection in tissue culture cells. J Gen Virol 74 (Pt 11):2347–2355. doi:10.1099/0022-1317-74-11-2347 7504071

[4] Dixon SJ, Lemberg KM, Lamprecht MR, Skouta R, Zaitsev EM, Gleason CE, Patel DN, Bauer AJ, Cantley AM, Yang WS, Morrison B 3rd, Stockwell BR. 2012. Ferroptosis: an iron-dependent form of nonapoptotic cell death. Cell 149:1060–1072. doi:10.1016/j.cell.2012.03.042 PMC3367386 22632970

[5] Zhao X, Zhang Y, Luo B. 2025. Ferroptosis, from the virus point of view: opportunities and challenges. Crit Rev Microbiol 51:246–263. doi:10.1080/1040841X.2024.2340643 38588443

[6] Niu S, Deng Y, Yang Y, Wang J, Fang C, Zhou Y, Dai H. 2025. A comprehension and systematic insight into the interaction between ferroptosis and virus infection: the implications of mechanisms and strategies. Virulence 16:2532806. doi:10.1080/21505594.2025.2532806 PMC12269692 40658446

[7] Dixon SJ, Stockwell BR. 2019. The hallmarks of ferroptosis. Annu Rev Cancer Biol 3:35–54. doi:10.1146/annurev-cancerbio-030518-055844 PMC12851585 41613499

[8] Yagoda N, von Rechenberg M, Zaganjor E, Bauer AJ, Yang WS, Fridman DJ, Wolpaw AJ, Smukste I, Peltier JM, Boniface JJ, Smith R, Lessnick SL, Sahasrabudhe S, Stockwell BR. 2007. RAS-RAF-MEK-dependent oxidative cell death involving voltage-dependent anion channels. Nature 447:864–868. doi:10.1038/nature05859 PMC3047570 17568748

[9] Feng F, He S, Li X, He J, Luo L. 2024. Mitochondria-mediated ferroptosis in diseases therapy: from molecular mechanisms to implications. Aging Dis 15:714–738. doi:10.14336/AD.2023.0717 PMC10917537 37548939

[10] Javadov S. 2022. Mitochondria and ferroptosis. Curr Opin Physiol 25:100483. doi:10.1016/j.cophys.2022.100483 PMC8944045 35342847

[11] Gao M, Yi J, Zhu J, Minikes AM, Monian P, Thompson CB, Jiang X. 2019. Role of mitochondria in ferroptosis. Mol Cell 73:354–363. doi:10.1016/j.molcel.2018.10.042 PMC6338496 30581146

[12] Wang J, Zhu J, Ren S, Zhang Z, Niu K, Li H, Wu W, Peng C. 2023. The role of ferroptosis in virus infections. Front Microbiol 14:1279655. doi:10.3389/fmicb.2023.1279655 PMC10706002 38075884

[13] Wang M-P, Joshua B, Jin N-Y, Du S-W, Li C. 2022. Ferroptosis in viral infection: the unexplored possibility. Acta Pharmacol Sin 43:1905–1915. doi:10.1038/s41401-021-00814-1 PMC8646346 34873317

[14] Shinya K, Ebina M, Yamada S, Ono M, Kasai N, Kawaoka Y. 2006. Avian flu: influenza virus receptors in the human airway. Nature 440:435–436. doi:10.1038/440435a 16554799

[15] Saunders-Hastings PR, Krewski D. 2016. Reviewing the history of pandemic influenza: understanding patterns of emergence and transmission. Pathogens 5:66. doi:10.3390/pathogens5040066 PMC5198166 27929449

[16] Letafati A, Taghiabadi Z, Ardekani OS, Abbasi S, Najafabadi AQ, Jazi NN, Soheili R, Rodrigo R, Yavarian J, Saso L. 2024. Unveiling the intersection: ferroptosis in influenza virus infection. Virol J 21:185. doi:10.1186/s12985-024-02462-3 PMC11321227 39135112

[17] Cheng J, Tao J, Li B, Shi Y, Liu H. 2022. Swine influenza virus triggers ferroptosis in A549 cells to enhance virus replication. Virol J 19:104. doi:10.1186/s12985-022-01825-y PMC9205082 35715835

[18] Huang J, Ma X, Liao Z, Liu Z, Wang K, Feng Z, Ning Y, Lu F, Li L. 2023. Network pharmacology and experimental validation of maxing Shigan decoction in the treatment of influenza virus-induced ferroptosis. Chin J Nat Med 21:775–788. doi:10.1016/S1875-5364(23)60457-1 37879795

[19] Ouyang A, Chen T, Feng Y, Zou J, Tu S, Jiang M, Sun H, Zhou H. 2024. The hemagglutinin of influenza A virus induces ferroptosis to facilitate viral replication. Adv Sci (Weinh) 11:e2404365. doi:10.1002/advs.202404365 PMC11497066 39159143

[20] Mazel-Sanchez B, Niu C, Williams N, Bachmann M, Choltus H, Silva F, Serre-Beinier V, Karenovics W, Iwaszkiewicz J, Zoete V, Kaiser L, Hartley O, Wehrle-Haller B, Schmolke M. 2023. Influenza A virus exploits transferrin receptor recycling to enter host cells. Proc Natl Acad Sci USA 120:e2214936120. doi:10.1073/pnas.2214936120 PMC10214170 37192162

[21] Zhang Y, Bergelson JM. 2005. Adenovirus receptors. J Virol 79:12125–12131. doi:10.1128/JVI.79.19.12125-12131.2005 PMC1211528 16160140

[22] Excoffon KJDA, Gansemer ND, Mobily ME, Karp PH, Parekh KR, Zabner J. 2010. Isoform-specific regulation and localization of the coxsackie and adenovirus receptor in human airway epithelia. PLoS One 5:e9909. doi:10.1371/journal.pone.0009909 PMC2845650 20361046

[23] Wold W, Ison M. 2013. Adenoviruses. Fields virology 2:1732–1767. doi:10.1128/microbiolspec.DMIH2-0020-2015

[24] Human Adenovirus Working Group. 2026. Human adenovirus working group. Available from: http://hadvwg.gmu.edu

[25] Braithwaite AW, Russell IA. 2001. Induction of cell death by adenoviruses. Apoptosis 6:359–370. doi:10.1023/a:1011338119712 11483860

[26] Lynch J III, Kajon A. 2016. Adenovirus: epidemiology, global spread of novel serotypes, and advances in treatment and prevention. Semin Respir Crit Care Med 37:586–602. doi:10.1055/s-0036-1584923 PMC7171713 27486739

[27] MacNeil KM, Dodge MJ, Evans AM, Tessier TM, Weinberg JB, Mymryk JS. 2023. Adenoviruses in medicine: innocuous pathogen, predator, or partner. Trends Mol Med 29:4–19. doi:10.1016/j.molmed.2022.10.001 PMC9742145 36336610

[28] Lion T. 2014. Adenovirus infections in immunocompetent and immunocompromised patients. Clin Microbiol Rev 27:441–462. doi:10.1128/CMR.00116-13 PMC4135893 24982316

[29] Orino K, Tsuji Y, Torti FM, Torti SV. 1999. Adenovirus E1A blocks oxidant-dependent ferritin induction and sensitizes cells to pro-oxidant cytotoxicity. FEBS Lett 461:334–338. doi:10.1016/s0014-5793(99)01443-x 10567722

[30] Dong W, Du K, Ding Y, Liu Y, Peng L, Wu C, Sun Y, Li Z, Niu Y. 2024. FAdV-4-induced ferroptosis affects fat metabolism in LMH cells. Vet Microbiol 293:110068. doi:10.1016/j.vetmic.2024.110068 38579482

[31] Pogorzelska A, Kuropka P, Stygar D, Michalczyk K, Chełmecka E, Zalejska-Fiolka J, Kublicka A, Janicka P, Bażanów B. 2024. Impact of human adenovirus 36 on embryonated chicken eggs: insights into growth mechanisms. Int J Mol Sci 25:2341. doi:10.3390/ijms25042341 PMC10889315 38397017

[32] Wang ZQ, Yu Y, Zhang XH, Floyd EZ, Cefalu WT. 2010. Human adenovirus 36 decreases fatty acid oxidation and increases de novo lipogenesis in primary cultured human skeletal muscle cells by promoting Cidec/FSP27 expression. Int J Obes (Lond) 34:1355–1364. doi:10.1038/ijo.2010.77 PMC3962839 20440297

[33] Liu C, Wu X, Bing X, Qi W, Zhu F, Guo N, Li C, Gao X, Cao X, Zhao M, Xia M. 2023. H1N1 influenza virus infection through NRF2-KEAP1-GCLC pathway induces ferroptosis in nasal mucosal epithelial cells. Free Radic Biol Med 204:226–242. doi:10.1016/j.freeradbiomed.2023.05.004 37146698

[34] Jansen SR, Poppinga WJ, de Jager W, Lezoualc’h F, Cheng X, Wieland T, Yarwood SJ, Gosens R, Schmidt M. 2016. Epac1 links prostaglandin E2 to β-catenin-dependent transcription during epithelial-to-mesenchymal transition. Oncotarget 7:46354–46370. doi:10.18632/oncotarget.10128 PMC5216803 27344171

[35] Cattani-Cavalieri I, Trombetta-Lima M, Yan H, Manzano-Covarrubias AL, Baarsma HA, Oun A, van der Veen MM, Oosterhout E, Dolga AM, Ostrom RS, Valenca SS, Schmidt M. 2024. Diesel exhaust particles alter mitochondrial bioenergetics and cAMP producing capacity in human bronchial epithelial cells. Front Toxicol 6:1412864. doi:10.3389/ftox.2024.1412864 PMC11306203 39118833

[36] Pei Z, Qin Y, Fu X, Yang F, Huo F, Liang X, Wang S, Cui H, Lin P, Zhou G, Yan J, Wu J, Chen Z-N, Zhu P. 2022. Inhibition of ferroptosis and iron accumulation alleviates pulmonary fibrosis in a bleomycin model. Redox Biol 57:102509. doi:10.1016/j.redox.2022.102509 PMC9614651 36302319

[37] Kistemaker LEM, Hiemstra PS, Bos IST, Bouwman S, van den Berge M, Hylkema MN, Meurs H, Kerstjens HAM, Gosens R. 2015. Tiotropium attenuates IL-13-induced goblet cell metaplasia of human airway epithelial cells. Thorax 70:668–676. doi:10.1136/thoraxjnl-2014-205731 25995156

[38] Rahmawati SF, Vos R, Bos IST, Kerstjens HAM, Kistemaker LEM, Gosens R. 2022. Function-specific IL-17A and dexamethasone interactions in primary human airway epithelial cells. Sci Rep 12:11110. doi:10.1038/s41598-022-15393-2 PMC9247091 35773318

[39] van Wetering S, van der Linden AC, van Sterkenburg MA, Rabe KF, Schalkwijk J, Hiemstra PS. 2000. Regulation of secretory leukocyte proteinase inhibitor (SLPI) production by human bronchial epithelial cells: increase of cell-associated SLPI by neutrophil elastase. J Investig Med 48:359–366. 10979241

[40] Zuo H, Trombetta-Lima M, Heijink IH, van der Veen CHTJ, Hesse L, Faber KN, Poppinga WJ, Maarsingh H, Nikolaev VO, Schmidt M. 2020. A-kinase anchoring proteins diminish TGF-β1/cigarette smoke-induced epithelial-to-mesenchymal transition. Cells 9:356. doi:10.3390/cells9020356 PMC7072527 32028718

[41] Audouy SAL, van der Schaaf G, Hinrichs WLJ, Frijlink HW, Wilschut J, Huckriede A. 2011. Development of a dried influenza whole inactivated virus vaccine for pulmonary immunization. Vaccine 29:4345–4352. doi:10.1016/j.vaccine.2011.04.029 21514345

[42] Lefkovits I, Cosenza H. 1979. 19 - Assay for plaque-forming cells, p 277–285. *In* Lefkovits I, Pernis B (ed), Immunological methods. Academic Press.

[43] Yang F, Pang B, Lai KK, Cheung NN, Dai J, Zhang W, Zhang J, Chan K-H, Chen H, Sze K-H, Zhang H, Hao Q, Yang D, Yuen K-Y, Kao RY. 2021. Discovery of a novel specific inhibitor targeting influenza A virus nucleoprotein with pleiotropic inhibitory effects on various steps of the viral life cycle. J Virol 95:e01432-20. doi:10.1128/JVI.01432-20 PMC8104107 33627391

[44] Danskog K, Mistry N, Årdahl C, Durbeej M, Forsell MNE, Lenman A, Arnberg N. 2025. Lactoferricin enables adenovirus infection of human skeletal muscle cells. Npj Viruses 3:62. doi:10.1038/s44298-025-00144-7 PMC12361444 40826223

[45] Schneider CA, Rasband WS, Eliceiri KW. 2012. NIH Image to ImageJ: 25 years of image analysis. Nat Methods 9:671–675. doi:10.1038/nmeth.2089 PMC5554542 22930834

[46] Zhang L, Yan H, Rahman MS, Mol-van der Veen CHJ, Covarrubias AM, Rafie K, Pendin D, Schmidt M, Dolga AM. 2025. Regulation of calcium signaling prevents neuronal death mediated by NIST DEP in xenoferroptotic cell death conditions. J Hazard Mater 488:137374. doi:10.1016/j.jhazmat.2025.137374 39892142

[47] Schindelin J, Arganda-Carreras I, Frise E, Kaynig V, Longair M, Pietzsch T, Preibisch S, Rueden C, Saalfeld S, Schmid B, Tinevez J-Y, White DJ, Hartenstein V, Eliceiri K, Tomancak P, Cardona A. 2012. Fiji: an open-source platform for biological-image analysis. Nat Methods 9:676–682. doi:10.1038/nmeth.2019 PMC3855844 22743772

[48] Yan H, Leshan Z, Daan PB. 2025. Exposure to diesel exhaust particles accelerated neuronal cell death in xenoferroptosis-susceptible cells. J Environ Sci 163. doi:10.1016/j.jes.2025.08.036 41887841

[49] Murray MB, Leak LB, Lee WC, Dixon SJ. 2023. Protocol for detection of ferroptosis in cultured cells. STAR Protoc 4:102457. doi:10.1016/j.xpro.2023.102457 PMC10432795 37556320

[50] Leon J, Borges CR. 2020. Evaluation of oxidative stress in biological samples using the thiobarbituric acid reactive substances assay. J Vis Exp. doi:10.3791/61122 PMC9617585 32478759

[51] Valente AJ, Maddalena LA, Robb EL, Moradi F, Stuart JA. 2017. A simple ImageJ macro tool for analyzing mitochondrial network morphology in mammalian cell culture. Acta Histochem 119:315–326. doi:10.1016/j.acthis.2017.03.001 28314612

[52] Chaudhry A, Shi R, Luciani DS. 2020. A pipeline for multidimensional confocal analysis of mitochondrial morphology, function, and dynamics in pancreatic β-cells. Am J Physiol Endocrinol Metab 318:E87–E101. doi:10.1152/ajpendo.00457.2019 PMC7052579 31846372

[53] Yang Wan Seok, SriRamaratnam R, Welsch ME, Shimada K, Skouta R, Viswanathan VS, Cheah JH, Clemons PA, Shamji AF, Clish CB, Brown LM, Girotti AW, Cornish VW, Schreiber SL, Stockwell BR. 2014. Regulation of ferroptotic cancer cell death by GPX4. Cell 156:317–331. doi:10.1016/j.cell.2013.12.010 PMC4076414 24439385

[54] Yang W.S., Stockwell BR. 2008. Synthetic lethal screening identifies compounds activating iron-dependent, nonapoptotic cell death in oncogenic-RAS-harboring cancer cells. Chem Biol 15:234–245. doi:10.1016/j.chembiol.2008.02.010 PMC2683762 18355723

[55] Zheng Y, Sun J, Luo Z, Li Y, Huang Y. 2024. Emerging mechanisms of lipid peroxidation in regulated cell death and its physiological implications. Cell Death Dis 15:859. doi:10.1038/s41419-024-07244-x PMC11589755 39587094

[56] Wiernicki B, Dubois H, Tyurina YY, Hassannia B, Bayir H, Kagan VE, Vandenabeele P, Wullaert A, Vanden Berghe T. 2020. Excessive phospholipid peroxidation distinguishes ferroptosis from other cell death modes including pyroptosis. Cell Death Dis 11:922. doi:10.1038/s41419-020-03118-0 PMC7591475 33110056

[57] Tsikas D. 2017. Assessment of lipid peroxidation by measuring malondialdehyde (MDA) and relatives in biological samples: Analytical and biological challenges. Anal Biochem 524:13–30. doi:10.1016/j.ab.2016.10.021 27789233

[58] Crisostomo L, Soriano AM, Mendez M, Graves D, Pelka P. 2019. Temporal dynamics of adenovirus 5 gene expression in normal human cells. PLoS One 14:e0211192. doi:10.1371/journal.pone.0211192 PMC6345434 30677073

[59] Elesela S, Lukacs NW. 2021. Role of mitochondria in viral infections. Life (Basel) 11:232. doi:10.3390/life11030232 PMC7998235 33799853

[60] Cervantes-Silva MP, Cox SL, Curtis AM. 2021. Alterations in mitochondrial morphology as a key driver of immunity and host defence. EMBO Rep 22:e53086. doi:10.15252/embr.202153086 PMC8447557 34337844

[61] Pila-Castellanos I, Molino D, McKellar J, Lines L, Da Graca J, Tauziet M, Chanteloup L, Mikaelian I, Meyniel-Schicklin L, Codogno P, Vonderscher J, Delevoye C, Moncorgé O, Meldrum E, Goujon C, Morel E, de Chassey B. 2021. Mitochondrial morphodynamics alteration induced by influenza virus infection as a new antiviral strategy. PLoS Pathog 17:e1009340. doi:10.1371/journal.ppat.1009340 PMC7920353 33596274

[62] Haas KM, McGregor MJ, Bouhaddou M, Polacco BJ, Kim E-Y, Nguyen TT, Newton BW, Urbanowski M, Kim H, Williams MAP, et al.. 2023. Proteomic and genetic analyses of influenza A viruses identify pan-viral host targets. Nat Commun 14:6030. doi:10.1038/s41467-023-41442-z PMC10533562 37758692

[63] Wang H, Wu X, Wu X, Liu J, Yan Y, Wang F, Li L, Zhou J, Liao M. 2020. Iron status is linked to disease severity after avian influenza virus H7N9 infection. Asia Pac J Clin Nutr 29:593–602. doi:10.6133/apjcn.202009_29(3).0019 32990620

[64] Lalueza A, Ayuso B, Arrieta E, Trujillo H, Folgueira D, Cueto C, Serrano A, Laureiro J, Arévalo-Cañas C, Castillo C, Díaz-Pedroche C, Lumbreras C, INFLUDOC group. 2020. Elevation of serum ferritin levels for predicting a poor outcome in hospitalized patients with influenza infection. Clin Microbiol Infect 26:1557. doi:10.1016/j.cmi.2020.02.018 32120038

[65] Lejeune N, Poulain F, Willemart K, Blockx Z, Mathieu S, Gillet NA. 2021. Infection of bronchial epithelial cells by the human adenoviruses A12, B3, and C2 differently regulates the innate antiviral effector APOBEC3B. J Virol 95:e0241320. doi:10.1128/JVI.02413-20 PMC8315923 33853956

[66] Qi L, Wang Y, Wang H, Deng J. 2020. Adenovirus 7 induces interlukin-6 expression in human airway epithelial cells *via* p38/NF-κB signaling pathway. Front Immunol 11:551413. doi:10.3389/fimmu.2020.551413 PMC7538593 33072092

[67] Musheshe N, Oun A, Sabogal-Guáqueta AM, Trombetta-Lima M, Mitchel SC, Adzemovic A, Speek O, Morra F, van der Veen CHJT, Lezoualc’h F, Cheng X, Schmidt M, Dolga AM. 2022. Pharmacological inhibition of Epac1 averts ferroptosis cell death by preserving mitochondrial integrity. Antioxidants (Basel) 11:314. doi:10.3390/antiox11020314 PMC8868285 35204198

[68] Wang R, Zhu Y, Ren C, Yang S, Tian S, Chen H, Jin M, Zhou H. 2021. Influenza A virus protein PB1-F2 impairs innate immunity by inducing mitophagy. Autophagy 17:496–511. doi:10.1080/15548627.2020.1725375 PMC8007153 32013669

[69] Varga ZT, Grant A, Manicassamy B, Palese P. 2012. Influenza virus protein PB1-F2 inhibits the induction of type I interferon by binding to MAVS and decreasing mitochondrial membrane potential. J Virol 86:8359–8366. doi:10.1128/JVI.01122-12 PMC3421771 22674996

[70] Yoshizumi T, Ichinohe T, Sasaki O, Otera H, Kawabata S, Mihara K, Koshiba T. 2014. Influenza A virus protein PB1-F2 translocates into mitochondria via Tom40 channels and impairs innate immunity. Nat Commun 5:4713. doi:10.1038/ncomms5713 25140902

[71] Matrosovich MN, Matrosovich TY, Gray T, Roberts NA, Klenk H-D. 2004. Human and avian influenza viruses target different cell types in cultures of human airway epithelium. Proc Natl Acad Sci USA 101:4620–4624. doi:10.1073/pnas.0308001101 PMC384796 15070767

[72] Kim S, Kim M-J, Park DY, Chung HJ, Kim C-H, Yoon J-H, Kim HJ. 2015. Mitochondrial reactive oxygen species modulate innate immune response to influenza A virus in human nasal epithelium. Antiviral Res 119:78–83. doi:10.1016/j.antiviral.2015.04.011 25930096

[73] Kroeker AL, Ezzati P, Coombs KM, Halayko AJ. 2013. Influenza A infection of primary human airway epithelial cells up-regulates proteins related to purine metabolism and ubiquitin-related signaling. J Proteome Res 12:3139–3151. doi:10.1021/pr400464p 23750822

[74] Kazer SW, Match CM, Langan EM, Messou M-A, LaSalle TJ, O’Leary E, Marbourg J, Naughton K, von Andrian UH, Ordovas-Montanes J. 2024. Primary nasal influenza infection rewires tissue-scale memory response dynamics. Immunity 57:1955–1974. doi:10.1016/j.immuni.2024.06.005 PMC11324402 38964332

[75] Yamada K, St Croix C, Stolz DB, Tyurina YY, Tyurin VA, Bradley LR, Kapralov AA, Deng Y, Zhou X, Wei Q, Liao B, Fukuda N, Sullivan M, Trudeau J, Ray A, Kagan VE, Zhao J, Wenzel SE. 2024. Compartmentalized mitochondrial ferroptosis converges with optineurin-mediated mitophagy to impact airway epithelial cell phenotypes and asthma outcomes. Nat Commun 15:5818. doi:10.1038/s41467-024-50222-2 PMC11237105 38987265

[76] Anand SK, Tikoo SK. 2013. Viruses as modulators of mitochondrial functions. Adv Virol 2013:738794. doi:10.1155/2013/738794 PMC3821892 24260034

[77] Lomonosova E, Subramanian T, Chinnadurai G. 2005. Mitochondrial localization of p53 during adenovirus infection and regulation of its activity by E1B-19K. Oncogene 24:6796–6808. doi:10.1038/sj.onc.1208836 16007153

[78] Perez D, White E. 2000. TNF-alpha signals apoptosis through a bid-dependent conformational change in Bax that is inhibited by E1B 19K. Mol Cell 6:53–63. doi:10.1016/S1097-2765(05)00013-4 10949027

[79] Han J, Modha D, White E. 1998. Interaction of E1B 19K with Bax is required to block Bax-induced loss of mitochondrial membrane potential and apoptosis. Oncogene 17:2993–3005. doi:10.1038/sj.onc.1202215 9881701

[80] Subramanian T, Vijayalingam S, Kuppuswamy M, Chinnadurai G. 2015. Interaction of cellular proteins with BCL-xL targeted to cytoplasmic inclusion bodies in adenovirus infected cells. Virology 483:21–31. doi:10.1016/j.virol.2015.04.015 PMC4516656 25965792

[81] Subramanian T, Vijayalingam S, Chinnadurai G. 2006. Genetic identification of adenovirus type 5 genes that influence viral spread. J Virol 80:2000–2012. doi:10.1128/JVI.80.4.2000-2012.2006 PMC1367173 16439556

[82] Jung M-S, Jin D-H, Chae H-D, Kang S, Kim S-C, Bang Y-J, Choi T-S, Choi K-S, Shin DY. 2004. Bcl-xL and E1B-19K proteins inhibit p53-induced irreversible growth arrest and senescence by preventing reactive oxygen species-dependent p38 activation. J Biol Chem 279:17765–17771. doi:10.1074/jbc.M305015200 14764594

[83] Schubert E, Mun K, Larsson M, Panagiotou S, Idevall-Hagren O, Svensson C, Punga T. 2024. Complex regulation of mitochondrial signaling by human adenovirus minor capsid protein VI. J Virol 98:e0035624. doi:10.1128/jvi.00356-24 PMC11265209 38837380

[84] Singh MK, Shin Y, Han S, Ha J, Tiwari PK, Kim SS, Kang I. 2024. Molecular chaperonin HSP60: current understanding and future prospects. IJMS 25:5483. doi:10.3390/ijms25105483 PMC11121636 38791521

[85] Qiu S, Zhong X, Meng X, Li S, Qian X, Lu H, Cai J, Zhang Y, Wang M, Ye Z, Zhang H, Gao P. 2023. Mitochondria-localized cGAS suppresses ferroptosis to promote cancer progression. Cell Res 33:299–311. doi:10.1038/s41422-023-00788-1 PMC10066369 36864172

[86] Chen Q, Sun L, Chen ZJ. 2016. Regulation and function of the cGAS-STING pathway of cytosolic DNA sensing. Nat Immunol 17:1142–1149. doi:10.1038/ni.3558 27648547

[87] Park H-S, Liu G, Liu Q, Zhou Y. 2018. Swine influenza virus induces RIPK1/DRP1-mediated interleukin-1 beta production. Viruses 10:419. doi:10.3390/v10080419 PMC6116029 30096906

[88] Merrill RA, Strack S. 2014. Mitochondria: a kinase anchoring protein 1, a signaling platform for mitochondrial form and function. Int J Biochem Cell Biol 48:92–96. doi:10.1016/j.biocel.2013.12.012 PMC3940257 24412345

[89] Zuo H, Han B, Poppinga WJ, Ringnalda L, Kistemaker LEM, Halayko AJ, Gosens R, Nikolaev VO, Schmidt M. 2018. Cigarette smoke up-regulates PDE3 and PDE4 to decrease cAMP in airway cells. Br J Pharmacol 175:2988–3006. doi:10.1111/bph.14347 PMC6016635 29722436

[90] Foret-Lucas C, Figueroa T, Bertin A, Bessière P, Lucas A, Bergonnier D, Wasniewski M, Servat A, Tessier A, Lezoualc’h F, Volmer R. 2023. EPAC1 pharmacological inhibition with AM-001 prevents SARS-CoV-2 and Influenza A virus replication in cells. Viruses 15:319. doi:10.3390/v15020319 PMC9965159 36851533

[91] Yang X, Long F, Jia W, Zhang M, Su G, Liao M, Zeng Z, Chen W, Chen J. 2023. Artesunate inhibits PDE4 leading to intracellular cAMP accumulation, reduced ERK/MAPK signaling, and blockade of influenza A virus vRNP nuclear export. Antiviral Res 215:105635. doi:10.1016/j.antiviral.2023.105635 37192683

[92] King CR, Gameiro SF, Tessier TM, Zhang A, Mymryk JS. 2018. Mimicry of cellular a kinase-anchoring proteins is a conserved and critical function of E1A across various human adenovirus species. J Virol 92:e01902-17. doi:10.1128/JVI.01902-17 PMC5874402 29367252

