# Supplementary material for: Respiratory viruses induce ferroptosis-like features in lung epithelial cells
Source: Microbiol Spectr. 2026 Aug 28;14(10):e01957-26. doi: 10.1128/spectrum.01957-26 (PMC13689485; doi:10.1128/spectrum.01957-26)
Supplement: Supplementary file 1 [file spectrum.01957-26-s0001.docx]

**Supplementary Information**

**Respiratory viruses induce ferroptosis-like features in lung epithelial cells**

Ana L. Manzano-Covarrubias, Alessandra Tosato, Christina HTJ Mol-van der Veen, Philip Ahmadyar, Jai Walia, Manon E. Woest, Jacqueline de Vries-Idema, Anke Huckriede, Amalia Dolga, Martina Schmidt, and Karim Rafie

**Supplementary Figures**

**Supplementary Fig. 1. H1N1 replication rate in lung epithelial cells**. Fluorescence images taken at a nominal magnification of 20x of (A) A549 and (B) BEAS-2B cells infected with H1N1/PR8 influenza virus at a MOI 1 fixed and stained at 4, 6, 8, 18 and 24 hpi with anti-influenza nucleoprotein (NP) antibody and counterstained with DAPI. Red: NP positive cells; Blue: nucleus (scale bar = 100 µm for A549 cells and 50 µm for BEAS-2B cells).

**Supplementary Fig. 2. HAdV-C5 replication rate in lung epithelial cells**. Fluorescence images taken at a nominal magnification of 20x of (A) A549 and BEAS-2B cells infected with HAdV-C5 virus at a MOI 1 fixed and stained at 24 hpi with anti-hexon antibody and counterstained with DAPI. Red: hexon positive cells; Blue: nucleus (scale bar = 100 µm).

**Supplementary Fig. 3. HAdV-C5 infection causes changes to mitochondria in A549 cells at later states of infection**. MitoTracker^TM^ Red FM fluorescence images taken at a nominal magnification of 60x of A549 cells (A) control and (B) after infection with HAdV-C5 at an MOI 1 at 24, 48, 60 and 72 hpi Red: mitochondria; Blue: nucleus (scale bar = 25 µm).

**Supplementary Figure 4. Apoptosis inhibitors do not rescue cell death caused by HAdV-C5 in alveolar epithelial (A549) and bronchial epithelial (BEAS-2B) cells.** MTT assay as measurement of cell viability. (A) A549 and (B) BEAS-2B cells at 72 hpi with HAdV-C5 (MOI = 1) and co-treated with Z-DEVD-FMK (10 µM) Z-VAD-FMK (10 µM) or Q-VD-Oph (10 µM). (C) A549 and (D) BEAS-2B cells after 72 h treatment with staurosporine (50 nM for A549, 5 nM for BEAS-2B) and co-treated with Z-DEVD-FMK (10 µM). (E) A549 and (F) BEAS-2B at 72 hpi with HAdV-C5 (MOI = 1) and co-treated with 50 or 100 µM Z-DEVD-FMK. Cell viability of the control groups were defined as 100% viability. Results are shown as percentage relative to the control (mean ± SD of technical replicates). Statistical analysis was performed using one-way ANOVA followed by Dunnett’s multiple comparisons test. *p < 0.05, **p < 0.01, ***p < 0.001, ****p < 0.0001. *50 µM Z-DEVD-FMK +/- HAdV represents two biological replicates, other results are of at least three biological replicates.
